# Comprehensive review of the resistance mechanisms of colorectal cancer classified by therapy type

**DOI:** 10.3389/fimmu.2025.1571731

**Published:** 2025-07-24

**Authors:** Janice M. Oh, Susan Kim, Carolyn Tsung, Eric Kent, Arad Jain, Samantha M. Ruff, Hongji Zhang

**Affiliations:** Department of Surgery, University of Virginia, Charlottesville, VA, United States

**Keywords:** colorectal cancer, chemotherapy, targeted therapy, immunotherapy, nanotechnology, radiation therapy, resistance mechanism

## Abstract

Colorectal cancer (CRC) is the fourth most diagnosed cancer and the second leading cause of cancer-related death in the United States. Despite advancements in treatment—including chemotherapy, targeted therapy with epidermal growth factor receptor antibodies, and immunotherapy with immune checkpoint inhibitors—many CRC cases exhibit intrinsic or acquired resistance to cancer treatment, leading to limited treatment efficacy and high recurrence rates. Resistance mechanisms encompass evasion of cell death pathways, alterations in drug metabolism, modulations of the tumor microenvironment, dysregulation of signaling pathways, and metabolic reprogramming. This review aims to provide a comprehensive overview of CRC resistance mechanisms categorized by therapy type, and to discuss emerging strategies, such as nanotechnology-based approaches, to address these therapeutic challenges.

## 1 Introduction

**Figure 1:**
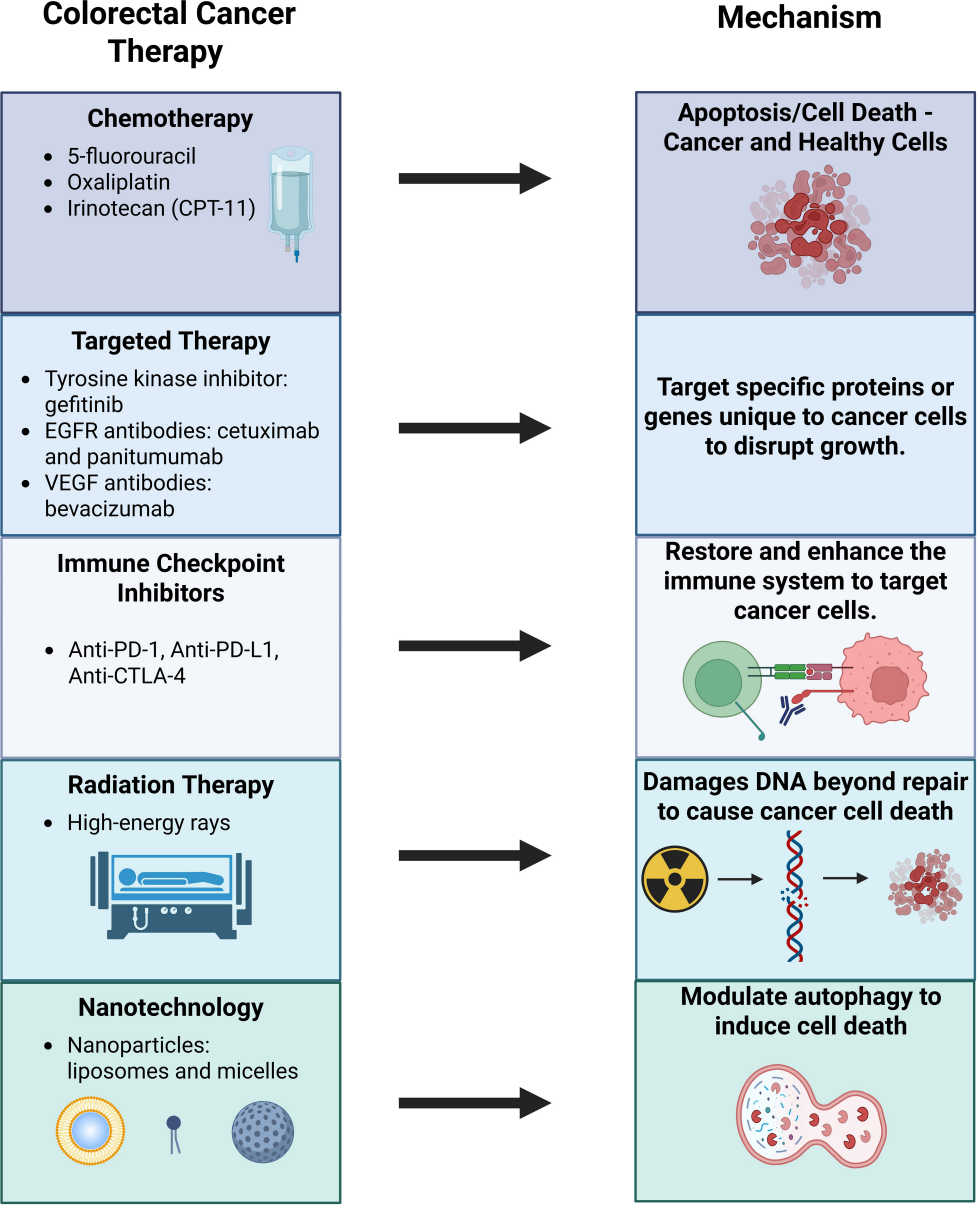
Current therapeutic treatments available for colorectal cancer.

Colorectal cancer (CRC) is the second leading cause of cancer-related deaths in the United States, with an estimated 53,010 deaths anticipated in 2024 (1). Over the years, multiple treatment modalities have been developed to combat colorectal cancer, including chemotherapy, targeted therapy, immunotherapy, and radiation therapy. Chemotherapy remains a cornerstone of treatment, with agents such as 5-fluorouracil (5-FU), oxaliplatin, and irinotecan (CPT-11) (**Figure 1**). Targeted therapy focuses on disrupting cancer cell processes critical for proliferation, differentiation, and migration. For instance, the epidermal growth factor receptor (EGFR), which is overexpressed in CRC, serves as a target for monoclonal antibodies like cetuximab and panitumumab, as well as tyrosine kinase inhibitors like gefitinib.

Immunotherapy has transformed cancer care by enhancing patient’s immune system to recognize and fight cancer. Immune checkpoint inhibitors (ICIs), such as monoclonal antibodies targeting programmed cell death protein-1 (PD-1), programmed death-ligand 1 (PD-L1), and cytotoxic T-lymphocyte-associated protein 4 (CTLA-4), have shown promise in CRC treatment (2). Radiation therapy, though more commonly used for rectal cancer rather than colon cancer, leverages high-energy rays to destroy cancer cells (3).

Despite these advancements, CRC outcomes remain suboptimal. Up to 29% of cases recur within five years after primary surgery, and only one third of CRC diagnoses are localized at the time of detection (4, 5). Consequently, while the overall five-year survival rate for CRC is 64%, this drops dramatically to 14% for patients with distant metastases (5). Moreover, the economic burden of CRC is significant, with treatment costs totaling $24.3 billion in 2020, ranking in second for cost of any cancer and encompassing 11.6% of all cancer treatment costs (6).

The persistently low survival rates and high recurrence of CRC can be attributed to cancer’s ability to develop resistance against existing therapies (7). Over 90% of deaths in cancer patients can be attributed to multidrug resistance (8). The development of resistance mechanisms to therapies confers decreased effectiveness of the administrated treatments, leading to decreased availability of viable treatment options and subsequent worsened prognosis (9). Such resistance can arise due to intrinsic factors, such as upregulation of intracellular defense pathways and alteration of drug targets. Resistance can also be due to extrinsic factors, such as changes in the tumor microenvironment that allow cancer cells to evade therapeutic treatments (10). Therefore, there is great prioritization of elucidating mechanisms of resistance in colorectal cancer in order to develop more effective treatments for colorectal cancer patients in the future. This review comprehensively presents the various mechanisms of resistance in CRC, categorized by types of treatments, and discusses promising treatment options that overcome these resistance mechanisms, including the advent of nanotechnology.

## 2 Mechanisms of chemotherapy resistance in CRC

### 2.1 Resistance due to modulation of cell death pathways

#### 2.1.1 Evasion of apoptosis

Apoptosis is a natural process of programmed cell death, allowing for the regulation of homeostasis through the maintenance of normal cells and embryonic development (11). Evasion of apoptosis is a hallmark of cancer, allowing cancer cell proliferation and resistance to chemotherapy (12). Various cancers have been shown to have increased expression of antiapoptotic proteins and suppression of proapoptotic proteins to resist treatments (13).

p53 is a crucial tumor suppressor that responds to a variety of stress signals, including DNA damage and oncogene activation. p53 protects genetic integrity and cell function by regulating various cellular responses, such as halting the cell cycle or inducing apoptosis (14). The *TP53* gene is mutated in 43% of colorectal cancers and many of the remaining CRC tumors have abnormal p53 functioning due to mutations in regulators of p53 (15). Such loss of p53 function has been shown to decrease levels of apoptosis in colorectal cancer cell lines, correlating with modulation of specific enzymes and contributing to resistance to 5-FU and oxaliplatin (16). Interestingly, Boyer et al. claimed that CPT-11 resistance was not shown to be related to p53 functioning status (16). Loss of p53 binding protein in colorectal cancer cell lines also leads to suppression of ATM-BHK2-P53 signaling. This causes cell proliferation and inhibition of apoptosis, leading to 5-FU resistance as well (17). Clinically, colorectal cancer patients in the N1 category with p53 mutations have had a weaker response to 5-FU treatment and shorter survival, compared to those with wild-type p53 tumors (18).

X-linked inhibitor of apoptosis protein (XIAP) suppresses apoptosis by inhibiting caspases (19). High-temperature requirement factor A1 (HtrA1) is a serine protease that regulates many cellular processes, such as apoptosis and proliferation. In SW480 colon cancer cell lines, HtrA1 was found to be downregulated, levels of XIAP were increased, and Akt activation increased, leading to cisplatin resistance (20). Likewise, in colon cancer cells and colon cancer xenograft models, Akt activation led to increased expression of XIAP and 5-FU resistance (21).

Bcl-2 family members were found to be involved in chemotherapy resistance as well. In human colorectal cancer cell lines, loss of proapoptotic protein Bax led to 5-FU resistance (22). Likewise, increased expression of antiapoptotic proteins Mcl-1 and Bcl-xL led to decreased activation of Bax and Bak, conferring resistance to oxaliplatin and MRK-003 (23). Colorectal cancer cell lines resistant to 5-FU demonstrated overexpression of non-coding RNA plasmacytoma variant translocation 1 (PVT1), which suppressed apoptosis. An increase in PVT1 correlated with increased levels of multidrug resistance proteins, including multidrug resistance-associated protein 1, p-glycoprotein, serine/threonine kinase mTOR, and apoptosis regulator Bcl-2 (24).

#### 2.1.2 Ferroptosis

Ferroptosis is an iron-dependent, non-apoptotic method of cell death, induced by reactive oxygen species and an imbalance of intracellular lipid redox. Glutathione peroxidase 4 (GPX4) plays a role reducing peroxidated phospholipids, preventing ferroptosis (25, 26). Thus, colorectal cancer cells that are resistant to oxaliplatin have shown increased levels of GPX4, allowing inhibition of ferroptosis. Specifically, the resistance correlates with the activation of the KIF20A/NUAK1/PP1β/GPX4 pathway (26).

Nuclear factor erythroid 2-related factor 2 (Nrf2) plays a key role in inhibiting ferroptosis by suppressing cellular iron reuptake, restricting the production of ROS, and upregulating solute carrier family 7 member 11 (SLC7A11), a cystine/glutamate antiporter (27). In human CRC 5-FU resistant cell lines, there were increased levels of Nrf-2, nuclear translocation, and binding to promotor due to hypomethylation of the Nrf2 promoter CpG islands. Activated Nrf2 leads to increased activity and protein expression of heme oxygenase-1 (HO-1), an Nrf2-regulated gene. 5-FU-resistant cells also produced more reactive oxygen species, leading to upregulation of DNA demethylase ten-eleven translocation 1 (TET1) (28).

Lipocalin-2 (LCN-2) is a glycoprotein that regulates iron homeostasis. Colon cancer cell lines *in vitro* and *in vivo* have demonstrated that LCN-2 is overexpressed and inhibits ferroptosis to confer resistance to 5-FU. LCN-2 decreases intracellular iron levels, increases GPX4 expression, and increases expression of xCT, a part of a cysteine glutamate antiporter (25).

### 2.2 Increased drug inactivation and reduced drug activation

Recent findings have also found resistance can be attributed to increasing chemotherapeutic drug inactivation or decreasing drug activation. Dihydropyrimidine dehydrogenase (DPD) catabolizes 5-FU into inactive metabolites (29). In CRC patients, high expression of DPD was associated with resistance to 5-FU (30).

Orotate phosphoribosyltransferase (OPRT), UMP kinase, and uridine monophosphate synthetase aid in converting 5-FU into active metabolites. These enzymes have been found to be downregulated in CRC cells with 5-FU resistance (16, 31, 32). Decreased levels of UMP kinase mRNA have been found in colorectal cancer hepatic metastases from patients as well (32).

### 2.3 Drug transport-based cellular mechanisms

High expression levels of p-glycoprotein drug efflux pump, a member of the ATP-binding cassette (ABC) transporter superfamily, have been associated with daunorubicin, doxorubicin, oxaliplatin, and 5-FU resistance in colorectal cancer (33–36). Interestingly, Ca2+-permeable transient receptor potential canonical protein 5 (TrpC5) has been described to induce p-gp expression in CRC cells (37). P-gp has also been found to be upregulated by interleukin-8 in doxorubicin-resistant CRC cells (38).

Breast cancer resistance protein (BCRP), another member of the ABC transporter superfamily, has been found to be overexpressed in CRC cells resistant to mitoxantrone, oxaliplatin, cisplatin, doxorubicin, and hydroxycampothecin (16, 39–42). BCRP has been found to be associated with c-MET/PI3K and JNK1 Signaling pathways in multidrug-resistant CRC (40, 42).

Elevated mRNA levels of multidrug resistance-associated protein 2 (MRP2), another member of the ABC transporter superfamily, were found in colorectal cancer patients and cells resistant to cisplatin and oxaliplatin (39, 43, 44). ABCB5 was also overexpressed in human CRC cells resistant to 5-FU (45).

Key transcription factor FOXM1 is overexpressed and promotes transcription of efflux transporter ABCC10 *in vitro* and *in vivo* for 5-FU-resistant colorectal cancer cells. Higher levels of ABCC10 also correlated with oxaliplatin resistance in multiple CRC cell lines. Clinically, CRC tissues had greater expression of FOXM1 and ABCC10. Increased ABCC10 levels positively correlated with relapse and metastasis in CRC patients as well (46, 47).

### 2.4 Changes in drug targets

Thymidylate synthase (TS), the target of 5-FU, was overexpressed in 5-FU-resistant HCT116 colon cancer cells and associated with poor survival (48). FOXM1 modulates expression levels of thymidylate synthase, contributing to resistance of 5-FU in colorectal cancer as well (49). 5-FU-resistant colon cancer cell lines showed increased chaperone protein HSP90 activity and upregulation of client protein Src, both of which led to increased TS expression (50).

Topoisomerase I is a target of SN-38, the active form of CPT-11. CPT-11 resistant CRC cell lines demonstrated downregulation of topoisomerase I mRNA and increased cellular export of SN-38 (16).

### 2.5 Tumor microenvironment

#### 2.5.1 Tumor-associated fibroblasts

Tumor-associated fibroblast (TAF)-derived CCL2 and downstream transcription factor Ets-1 are associated with TAF-induced fibroblast growth factor receptor 4 (FGFR4) upregulation in colorectal cancer cell lines. Specifically, FGFR4 aids in TAF-induced epithelial-mesenchymal transition (EMT). FGFR was also involved in the expression of anti-apoptotic proteins c-FLIP and Bcl-2 in colon cancer cells. These mechanisms confer resistance to chemotherapeutics such as 5-FU and oxaliplatin (51, 52). Additionally, cancer-associated fibroblasts (CAFs) were found to secrete proinflammatory cytokines, leading to resistance of CRC to oxaliplatin (53). TAFs can play a role in hypoxic conditions to secrete TGF-β2 and HIF-1α, which can activate hedgehog transcription factor *GLI2* expression and confer resistance to FOLFOX (5-FU and oxaliplatin) (54). Snail, a transcriptional repressor, was overexpressed in CRC cells and induced transdifferentiation of 3T3 fibroblasts to TAFs, endowing resistance to 5-FU and paclitaxel. This process involved chemokine C-C motif ligand-1 (CCL1) in Snail-expressed 3T3 fibroblasts activating TGF-β and NF-κB signaling pathways (55).

#### 2.5.2 Tumor-associated macrophages

Activated tumor-associated macrophages (TAMs) were found to secrete ornithine decarboxylase and trigger the JNK-caspase-3 pathway, conferring resistance to 5-FU in CRC (56). Tumor-associated macrophages were shown to upregulate chaperone protein glucose-regulated protein 78 and increase membrane translocation of ABC transporter multidrug-resistance protein 1 (MDR1). This allowed increased drug efflux and therefore resistance to 5-FU in colorectal cancer cells (57). Transcriptomics revealed that chemotherapy resistance in colorectal cancer liver metastasis is associated with infiltration of neutrophils and monocytes (58). Interleukin 6 (IL-6) produced by TAMs were found to induced CRC chemoresistance by regulating the IL6R/STAT3/miR-204-5p axis (59).

### 2.6 Other mechanisms

Cullin4 family member CUL4B, a protein involved in cell proliferation and cell cycle progression, has been found to modulate epithelial-mesenchymal transition (EMT) and confer resistance to oxaliplatin in CRC (60).

Dual serine/threonine and tyrosine protein kinase has been found to be involved in regulating TGF-β-induced EMT as well, leading to resistance to oxaliplatin in CRC cells (61).

Sex-determining region Y-box2 (SOX2), a transcriptional master regulator, was found to activate MRP2, β-catenin, and Beclin1 expression, influencing autophagy signaling. Activation of autophagy increases levels of stemness, EMT, and resistance to CPT-11 (62).

Fibroblasts were found to produce exosomal Wnts that reprogram CRC cells into cancer stem cells, conferring resistance to oxaliplatin and 5-FU (63). Resistance to oxaliplatin is induced in CRC by exosome-delivered circular RNA, which promotes glycolysis (64).

## 3 Mechanisms of targeted therapy resistance in CRC

### 3.1 Changes in drug targets of EGFR and EGFR ligands

CRC has been shown to resist cetuximab through mutations on the EGFR extracellular domain, including S492R, R198, R200, R451C, and K467T (65–67). Lower expression of EGFR ligands epiregulin and amphiregulin were correlated with less efficacy of cetuximab treatment in mCRC patients (68, 69).

### 3.2 Dysregulated activation of alternative receptors: IGF-1R, IGF-2R, HER2, and HER3

Insulin-like growth factor 1 receptor (IGF-1R), IGF-2R, human epidermal growth factor receptor 2 (HER2), HER3, and the hepatocyte growth factor/mesenchymal-epithelial transition factor (HGF/MET) pathway are involved in the activation of the PI3K/Akt and RAS/RAF/MEK/ERK pathways. Resistance to anti-EGFR monoclonal antibodies can develop from modulations to these pathways (70). For example, elevated IGF-1R activation was correlated with lower response rates in CRC patients treated with cetuximab (71). Overexpression of IGF2 was found to be responsible for cetuximab resistance in patient-derived xenografts from CRC liver metastases and CRC cell lines (72).

Loss of mismatch repair gene mutL homolog 1 (MLH1) has been shown to activate HER2/PI3K/Akt signaling to induce cetuximab resistance in colon cancer (73). HER2 activating mutations increased MAPK signaling, inducing resistance to cetuximab and panitumumab in CRC cell lines (74, 75). Additionally, amplification of *HER2* and heregulin upregulation have been shown to activate HER2 and ERK 1/2 signaling, leading to cetuximab resistance in various cancer cell lines. This was corroborated by clinical data, which demonstrated that CRC patients resistant to cetuximab had *HER2* amplification or elevated levels of circulating heregulin (76).

### 3.3 Modulation of the MET and KRAS

MET activation has been found to confer resistance to cetuximab and panitumumab in CRC (77). Additionally, a study on mCRC patient tumor sections revealed that tumors with KRAS mutations were resistant to panitumumab (78).

### 3.4 Tumor microenvironment

#### 3.4.1 Tumor-associated fibroblasts and cancer stem cells

Hepatocyte growth factor, secreted by TAFs, was found to bind to MET receptors and lead to the activation of MAPK and Akt signaling, conferring resistance to cetuximab in colon cancer stem-like cells (79).

Through genomic and transcriptomic analysis on CRCs, cetuximab resistance was found to be associated with NF1 and non-canonical RAS/RAF aberrations. However, non-genetic mechanisms of resistance to cetuximab were also found, via TAF-mediated stromal remodeling and secretion of various growth factors, including FGF1, FGF2, HGF, TGF-β1 and TGF-β2 (80).

Colon cancer stem cells have been found to be resistant to cetuximab by inducing anti-apoptotic signaling involving PP2A, p38MAPK, MAPKAPK2, and Hsp27 (81).

#### 3.4.2 Angiogenesis/the VEGF/VEGFR pathway

Anti-VEGFR treatments have been shown to induce hypoxia in the tumor microenvironment (TME), inducing extracellular matrix (ECM) remodeling and overexpression of hyaluronic acid in colorectal liver metastasis mouse models, conferring resistance to anti-VEGF therapy (82).

Elevated VEGF expression levels were associated with cetuximab resistance in mCRC patients (83). Placental growth factor (PlGF), a VEGFR1 ligand, was overexpressed in CRC patients resistant to antiangiogenic treatments (84). Additionally, elevated levels of angiopoietin-2 (ANG2), which works with VEGF to regulate vascular remodeling, were found in mCRC patients with weaker responses to bevacizumab (85).

#### 3.4.3 Metabolic reprogramming

Metabolic reprogramming is another mechanism of resistance for CRC. For example, in human colorectal carcinomas, it has been found that TNF receptor-associated protein1 (TRAP1) regulates phosphofructokinase 1 (PFK1) to increase levels of glycolysis, leading to resistance to cetuximab (86). Stiffening of the extracellular matrix was found to activate hepatic stellate cells and fatty acid secretion that stimulated the fatty acid oxidation pathway in colon cancer cells, leading to resistance to cetuximab (87). Expression of proteins involved in fatty acid metabolism, such as fatty acid binding protein and heat shock protein 27, have resulted in cetuximab resistance in CRC cells via anti-apoptotic effects as well (88).

### 3.5 Other mechanisms

Transcription factor Homeobox B9 (HOXB9) induced bevacizumab resistance in CRC mouse models (89).

Mutations of ARID1A, an epigenetic regulator, in colorectal cancer patients were found to confer resistance to cetuximab rather than bevacizumab, with a relation to the EGFR/MAPK pathway (90).

## 4 Mechanisms of immunotherapy resistance in CRC

### 4.1 Altered expression of HLA complexes

One study revealed that a microsatellite-unstable colorectal cancer patient with mutated β2-microglobulin (or HLA class I heavy chain) had demonstrated resistance to anti-PD-1 mAb treatment (91). In agreeance with this finding, a large-scale genomic analysis revealed that *B2M* and *HLA* mutations were prevalent in MSI-high CRC primary tumor samples, providing a means of immunotherapeutic resistance (92). High microsatellite (MSI-H) instability CRC tumors were also found to frequently have mutated *NLRC5* and *RFX5* genes, which regulate HLA Class I gene transcription (92, 93).

### 4.2 Tumor microenvironment

#### 4.2.1 Tumor-associated fibroblasts

In CRC mouse models, it was shown that TGF-β elevation in the tumor microenvironment confers resistance to anti-PD-1 therapy via inhibition of T-cell infiltration and decreased levels of type 1 T-helper cell phenotype (94).

NADPH oxidase 4 (NOX4) is involved in transdifferentiating fibroblasts to myofibroblasts. NOX4 was found to be upregulated in colon adenocarcinoma tumor samples, which was associated with elevated levels of myofibroblastic-CAFs and a possible mechanism for immunotherapeutic resistance (95).

#### 4.2.2 Myeloid-derived suppressor cells

Granulocyte myeloid-derived suppressor cells (g-MDSC’s) are recruited by IL-8, GM-CSF, TNF-alpha, YAP1, CXCR2, and CCL2, conferring tumor immunotherapy resistance to CRC (96–99).

Mononuclear MDSCs (M-MDSCs) have been shown to be implicated in resisting immune checkpoint inhibitors (anti-CTLA-4 and anti-PD-1) and are associated with receptor tyrosine kinase (KIT) signaling in CRC (100).

Mutation of KRAS was shown to repress interferon regulatory factor 2 (IRF2), leading to CXCL3 upregulation in CRC mouse models. CXCL3 binds to CXCR2 on myeloid-derived suppressor cells and aids in the migration of these cells into the tumor microenvironment, conferring resistance to anti-PD-1 (101).

### 4.3 Other mechanisms

It is possible that microsatellite stable CRC tumors are more resistant to immunotherapy compared to MSI tumors due to having fewer neoantigens (102).

Abnormal expression of group 3 innate lymphoid cells (ILC3) was found to confer changes to the gut microbiota and resistance to anti-PD-1 immunotherapy in CRC mice and patients. This was found to be due to dysregulated major histocompatibility complex class II-mediated communication between ILC3 and T cell (103).

## 5 Mechanisms of radiotherapy resistance in CRC

### 5.1 DNA damage repair

CRC tumor cells have been shown to display radioresistance from multiple mechanisms such as increased DNA damage repair and escape from apoptosis. For example, CRC cells exhibited upregulation of DNA repair proteins such as RAD50 and DNA-PK, which are critical for the homologous recombination and non-homologous end-joining pathways, respectively. This upregulation allowed for the correction of radiation-induced DNA damage to enable cancer cell survival (104).

### 5.2 Cancer stem cells

Radiation can also promote the activation of cancer stem cells (CSC), cells within the tumor microenvironment that exhibit stem cell process of self-renewal and differentiation which promotes cancer growth and metastases (104). Interestingly, cancer stem cells (CSC) have also been shown to possess enhanced DNA repair capabilities and robust antioxidant defenses, allowing them to survive radiation-induced damage more effectively than non-stem cancer cells (105).

### 5.3 Tumor microenvironment

Radiation exposure-induced chronic inflammation, fibrosis, vascular damage, and immunosuppression in the tumor microenvironment have all been shown to facilitate a pro-tumorigenic niche as well as radiotherapy resistance (106). For example, the release of interleukin-1α in murine rectal cancer models caused the differentiation of tumor-associated fibroblasts into more pro-tumorigenic fibroblasts and reduced the anti-tumor effect of radiotherapy (107). Additionally, the establishment of hypoxic tumor microenvironments and accumulation of hypoxia-inducible factor (HIF-1) have been mechanisms that resist radiotherapy and promote CRC survival (108).

## 6 Shared mechanisms of resistance in CRC

**Figure 2:**
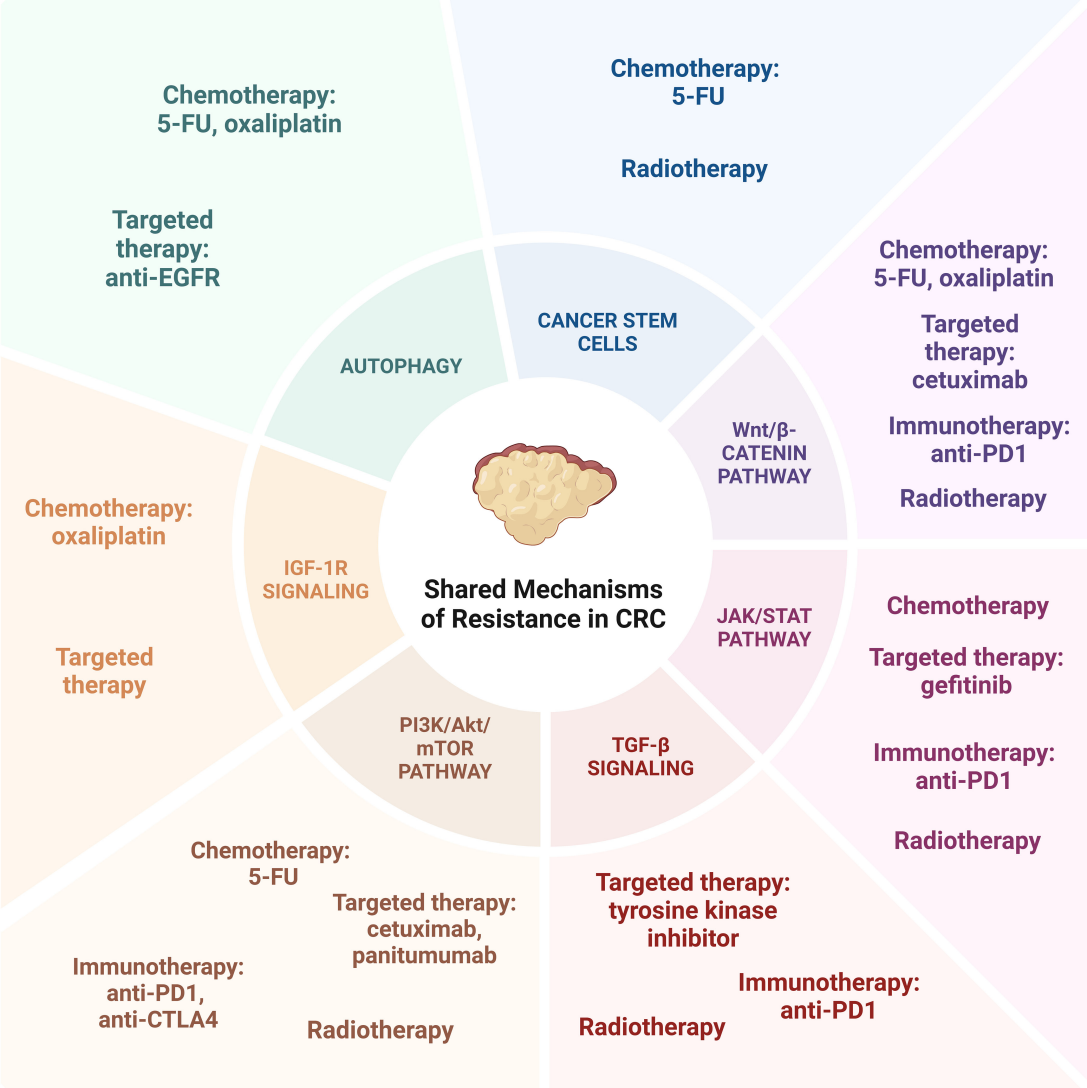
Shared Mechanisms of resistance in CRC.

While the modalities of colorectal cancer treatment each confer their own mechanisms of resistance, there are multiple shared pathways between treatment options that work in tandem to confer therapeutic resistance (**Figure 2**).

### 6.1 Autophagy

Autophagy, a process through which a cell recycles its own degraded products to maintain its own survival, has been implicated in treatment resistance, with cancer cells employing this process to redirect their cellular building blocks toward their rapidly proliferating cells. Several studies have demonstrated how the over-expression of key autophagy related genes lead to resistance, allowing rapidly proliferating cells to survive in the face of treatment. Wang et al. established that a long non-coding RNA SNHG6 sponged miR-26a-5p to target ULK1, initiating autophagosome formation. Ultimately, this leads to inhibition of 5-FU induced apoptosis and treatment resistance, which was demonstrated *in vitro* and *in vivo* (109). Similarly to SNHG6, Zhang et al. demonstrated oxaliplatin resistance may be conferred to a similar sponging mechanism, with circHIPK3 sponging MiR-637 to mediate autophagy. They were able to demonstrate this *in vitro*, with increased expression of circHIPK3 in oxaliplatin resistant cell lines, but not in 5-FU resistant cell lines. They then confirmed by measuring circulating circHIPK3 in patient samples, with higher levels detected in patients who were non-response to oxaliplatin (110).

Outside of chemotherapy, these patterns have been seen with anti-EGFR therapy as well. Guo et al. demonstrated in patients with advanced colorectal cancer treated by either anti-EGFR inhibition or without, the expression of autophagy proteins Beclin-1 and LC3 were not only associated with advanced colorectal cancer, but those with higher expression of these proteins experienced worse objective response rates, and shorter progression free survival. These were not detected in the cohort that did not receive anti-EGFR therapy, suggesting a connection between therapeutic resistance (111). Koustas et al. further confirmed the overexpression of Beclin-1 and its association with poorer survival was paralleled in patients with CRC undergoing chemotherapy, regardless of stage or mutational status (112).

### 6.2 Cancer stem cells

In addition to upregulation of autophagic mechanisms, treatment-resistant cancers have also been shown to demonstrate stem-like signatures (113). Colorectal cancer specifically has been shown to upregulate several stem cell markers, including BMI1, Nanog, and CD44 (114). CD133 in particular has been frequently explored in colorectal cancer. CD133 positive cells have been shown to stay undifferentiated for over a year, possess multilineage differentiation potential, and have confirmed they experience loss of CK20 expression, which is a marker of epithelial cell differentiation (115, 116). CD133 positive cells have also been implicated in initiation of tumor growth that is phenotypically similar to the original, suggesting its role in tumoral proliferation from lingering subpopulations after treatment. Furthermore, the presence of CD133 has been correlated with resistance to chemotherapy in multiple cancer types, including 5-FU resistance in colorectal cancer, hepatocellular carcinoma, and glioblastoma multiforme (117–119).

These cancer stem cells employ pro-survival mechanisms like advantages in enhanced DNA repair, anti-apoptotic signaling mechanisms, intrinsic drug efflux mechanisms, and the ability to exist in quiescent states, all of which lend to escaping therapeutic effect. Particularly the intrinsic ability to exist in quiescent states has demonstrated efficacy in resistance to chemotherapy and radiotherapy. As 5-FU-based chemotherapy relies on active DNA replication to take effect, this quiescent non-replicating state prevents 5-FU from its intended activity. Similarly, radiotherapy largely acts on the premise of DNA damage of rapidly proliferating cells, but when quiescent, these cells are less sensitive to these insults (120).

### 6.3 Persistent activation of oncogenic/bypass signaling & downstream signaling pathways

By far, cancer cells have adapted to confer resistance via multiple signaling pathways, either through persistent activation of oncogenes, bypassing regulatory signaling checkpoints, or upregulating downstream pathways. There are several well understood pathways that employ this strategy known thus far.

#### 6.3.1 Wnt/β-catenin pathway

Wnt/β-catenin pathway is altered in up to 90% of patients with colorectal cancer (121). The Wnt/β-catenin signaling pathway is critical in pro-survival signaling leading to self-renewal and growth, and interactions with other survival pathways such as STAT, MAPK, and PI3K/Akt, which is implicated in treatment resistance to multiple modalities of therapy, such as chemotherapy, targeted therapy such as cetuximab, anti-PD1 immune checkpoint inhibitors, as well as radiotherapy (122).

Several studies have demonstrated that blocking of Wnt/β-catenin related pathways lead to increased chemotherapy induced apoptosis, increasing sensitivity to chemotherapy (123, 124). This effect has been seen with 5-FU and oxaliplatin chemotherapies, through knock down of aquaporin-5 and MMP7 respectively (37, 125, 126). Additionally, chemotherapeutic resistance has been implied to occur through Wnt/B-catenin associated weakening of drug efflux mechanisms, either through suppression of MDR1 or weakening of the ABC1 efflux pump (37, 127).

In addition to chemotherapeutic resistance, the Wnt/β-catenin pathway has been implicated in resistance to other systemic therapies. Due to the close relationship of Wnt and EGFR, EGFR can form a complex with B-catenin to activate Wnt, and the Wnt ligand can activate Frizzled to lead to EGFR resistance, as seen with the development of targeted therapy resistance to cetuximab (128). This relationship has been explored by Lu et al. through whole exome sequencing and transcriptional profiling of cetuximab-resistant colorectal tumors; lncRNA MIR100HG and its derived miRs were overexpressed, with concomitant increase in Wnt signaling. Inhibition of Wnt in these resistant cells restored cetuximab sensitivity (129).

Furthermore, Wnt/β-catenin pathway has been implicated in the modulation of the tumor microenvironment leading to a less immunogenic landscape for immune checkpoint inhibitors to act. Wnt signaling reduces the frequency of tumor infiltrating T cells, leading to decreased ICI efficacy. However Xiao et al. demonstrated by blocking Wnt/β-catenin pathways improves CD8 T cell infiltration, rendering a more favorable environment for ICI, and enhancing the efficacy of anti-PD1 therapy (130, 131). Conversely, by upregulating APC, which upregulates Wnt signaling, it has been negatively correlated with the frequency of T cell infiltration (130).

Outside of systemic therapies, the Wnt/β-catenin pathway has also been implicated in radiotherapy resistance. Specifically, radiotherapy resistance is often related to an adaptation to the hypoxic environment conferred by the radiation insult, primarily through Wnt/β-catenin signaling pathway. Wnt increases β-catenin post-radiation leading to dedifferentiation of cancer stem cells. This subsequently activates Notch pathway, increasing the proportion of cells in S phase. Through inhibition of Notch and Wnt/β-catenin, Wang et al. demonstrated the down-regulation of SOX2, a stemness-associated gene, and resultant decrease of mass forming liver cancer stem cells (132). Similar *in-vitro* studies have not been conducted in colorectal cancer, however the underlying mechanism of Wnt/β-catenin signaling contributing to radiotherapy resistance in colorectal cancer has been explored, and dysregulated Notch or Wnt/β-catenin signaling has been demonstrated (133).

#### 6.3.2 JAK/STAT pathway

While the Wnt/β-catenin pathway has been explored significantly, there are other contributing signaling pathways to therapeutic resistance that are explored in the literature. JAK/STAT is of note, also conferring multi-therapeutic resistance. JAK and STAT regulate cell survival and differentiation, acting at the convergence point of multiple cellular pathways. In colorectal cancer, IL-6 induced phosphorylation results in constitutive activation of STAT3, which was positively correlated with resistance to chemoradiotherapy. Inhibition of STAT3 also reversed *in vitro* and *in vivo* resistance, further suggesting the association with resistance (134, 135). STAT3 upregulation also contributes to anti-EGFR targeted therapy resistance, specifically gefitinib, through overexpression of nuclear PKM2 (136).

Similar to other signaling pathways, JAK/STAT also modulates the tumor microenvironment, leading to variable immune checkpoint inhibitor efficacy and resistance. This is pronounced through the interferon-gamma (IFN-y) pathway. Downregulation or mutation of components of the JAK/STAT pathway have demonstrated blockade of IFN-y signaling and tumoral non-response to IFN-y, suggesting its involvement in the anti-tumor response (137, 138). Furthermore, JAK1 and JAK2 inactivation has been observed in high tumor mutational burden colon cancers that were non-responsive to anti-PD1 therapy, suggesting the role IFN-y plays in anti-PD1 resistance (139).

#### 6.3.3 TGF-β signaling

TGF-β has additionally been implicated in conferring treatment resistance through separate pathways, particularly by promoting epithelial to mesenchymal transition. This EMT transition by TGF-β has been identified in non-colon cancer cell lines as well as in colorectal cancer through disruption of MED12, and transcription regulation (140, 141). Through MED12 deletion, the MEK/ERK pathway is activated, restoring downstream MAPK pathway inhibition, overcoming tyrosine kinase inhibitor resistance in CRC (140).

Chemotherapy can also activate TGF-β pathways (142). By inhibiting these associated pathways through PAR2 inhibition, the proliferation of chemoresistance cells is reduced and cancer cell death is more abundant (143).

TGF-β signaling has also been implicating in attenuating the anti-tumor potential of the immune microenvironment through immune evasion (94, 144). This has been demonstrated in murine models with low mutational burden; T cell rejection and TGF-β activation led to decreased ICI benefit. However, by TGF-β signaling alone promoted enough T cell infiltration to re-sensitize to anti-PD1 therapies (145). This phenomenon crosses over into radiotherapy resistance, where TGF-β is thought to play a role in modulating treatment sensitivity and immune cell infiltration. Even in poorly immunogenic murine carcinoma models, TGF-β neutralization during radiation demonstrated an ability to generate a robust CD8 response that regressed irradiated tumor beds (146). Furthermore, TGF-β1 gene deletions or inhibition of TGF-β signaling in breast and glioblastoma models demonstrated increased radiation sensitivity, though this has yet to be demonstrated in colorectal cancer (147, 148).

#### 6.3.4 PI3K/Akt/mTOR pathway

An additional pathway implicated in multi-treatment resistance is the PI3K/Akt/mTOR pathway. 10-18% of patients with metastatic colorectal cancers carry PI3K mutations. Additionally, 20-40% of patients have loss of function mutations of PTEN, which is a suppressor of PI3K/Akt pathway. Ultimately, this results in constitutive activation of PI3K/Akt pathway, resulting in continued tumoral growth and resistance to EGFR blockade (149, 150). This clinically has translated to resistance to the EGFR inhibitors used as targeted therapy in metastatic colorectal cancer. Sartore-Bianchi et al. demonstrated these mutations resulted in a lack of response to cetuximab and panitumumab, leading to shorter progression free survival and poorer overall survival (151–153).

This phenomenon is paralleled in immune checkpoint inhibitor resistance. Loss of PTEN additionally leads to upregulation of PD-L1, leading to resistance to loss of CD8 T-cell infiltration, transforming the immune landscape of tumors and decreasing the efficacy of T-cell mediated immunotherapeutic agents (154, 155). As previously stated, PTEN is a negative regulator of PI3K/Akt/MTOR, therefore inhibition of this pathway has demonstrated improved response to anti-PD1 and anti-CTLA4 therapies, particularly in MSI-H and deficient MMR colorectal tumors (156).

The PI3K/Akt pathway additionally ties back to many of the other shared mechanisms of resistance, notably demonstrated through mechanisms of chemotherapeutic resistance. PIK3A mutations have been shown to increase PI3K/Akt signaling in LGR5+ CRC stem cells, leading to increased rates of proliferation and survival. This was further corroborated by the finding that CRC patients with the PIK3A mutation showed worse response to first-line chemotherapeutics (FOLFOX and XELOX) compared to those without mutations (157). Specifically, Hu et al. demonstrated that IL-6-induced activation of the JAK2/BECN1 pathway ultimately regulates PI3KC3 complex formation, and that this complex is a predictive marker for poor CRC prognosis and chemotherapeutic response (158). Separately, Liu et al. have demonstrated microRNAs (miR-135b and miR-182) were upregulated in 5-FU resistance cell lines, resulting in increased drug resistance, proliferation, and decreased apoptosis. They further clarified that this was through altered expression of ST6GALNAC2 which acts as a regulator of the PI3K/Akt pathway. By inhibiting this pathway, chemosensitivity to 5-FU was restored in two separate colorectal cell lines, HCT8 and LoVo (159).

PI3K/Akt pathways are implicated in radiotherapy resistance as well. As radiation generates oxidative stress, mTOR is activated, leading to intracellular signaling promoting cell survival. This relationship between radiotherapeutic resistance and the PI3K/Akt pathway was confirmed after utilizing NVP-BEZ235, a mTOR inhibitor, enhancing the radiosensitivity of previously resistant prostate cancer cells (160, 161). This was further explored in murine intestinal models, resulting in a similar pathway of oxidative stress and mTOR activation via PI3K/Akt (162).

#### 6.3.5 IGF-1R signaling

Finally, more novel shared mechanisms of resistance are coming to light as research advances. Codony-Servat et al. have demonstrated how nuclear IGF-1R can predict resistance to chemotherapy and targeted therapy in metastatic colorectal cancer. In their study, nuclear IGF-1R expression was detected by immunohistochemistry in 470 patients with metastatic colorectal cancer, with higher expression in tumors from patients that had undergone treatment. Furthermore, there was a significant correlation between IGF-1R expression and poorer overall survival. When evaluating *in vitro*, resistant cell lines also displayed higher IGF-1R expression, and this expression and nuclear localization was able to be induced following treatment with oxaliplatin (163).

## 7 Approaches to overcome resistance

The rise of resistance to standard CRC therapies has necessitated the development of innovative strategies to enhance treatment efficacy (**Table 1**). Combination therapy has shown promise in overcoming drug resistance in CRC. For example, the phytochemical apigenin enhances CRC cell sensitivity to 5-FU by inhibiting expression of 5-FU target thymidylate synthase and promoting 5-FU-mediated apoptosis (164). Mouse models for CRC genetic aberrations have demonstrated resistance to anti-VEGF antibodies due to tumor-infiltrating neutrophils that overexpresses angiogenesis-related Bv8/PROK2. Elevated plasma Bv8/PROK2 levels correlated with poor overall survival patients by suppressing these tumor-infiltrating neutrophils and the related angiogenic processes, restoring sensitivity to anti-VEGF therapies (165). Similarly, the gut microbiota member *Fusobacterium nucleatum* and its product succinic acid confer resistance to anti-PD-1 mAb by inhibiting the cGAS-interferon-β pathway, thereby impairing CD8+ T cell-mediated anti-tumor immunity in the CRC. Administering metronidazole to decrease *Fusobacterium nucleatum* levels re-sensitized CRC tumors to anti-PD-1 mAb treatment (166). Resistance mediated by MGAT3-modulated N-glycosylation, which suppresses IF-γ receptor α (IFNγRα) expression in CRC cells, was overcome by all-trans retinoic acid-induced MGAT3 expression (167). Additionally, YTH N (6)-methyladenosine RNA binding protein 1 (YTHDF1) drives resistance to anti-PD-1 therapy by modulating the m (6)A-p56-CXCL1/CXCR2 axis, a mechanism reversed through CRISPR or nanoparticle-mediated YTHDF1 silencing (168).

**Table 1:** Examples of strategies to overcome therapy resistance.

Overcoming strategy/Type of therapy resistance	Chemotherapy	Targeted therapy	Immunotherapy	Radiotherapy
Combination therapy	Apigenin can inhibit the 5-FU target thymidylate synthase and promote 5-FU mediated apoptosis	Suppression of tumor-infiltrating neutrophils to increase Bv8/PROK2 expression can improve sensitivity to anti-VEGF antibodies	Fusobacterium nucleatum can inhibit cGAS-interferon-β pathway and impair CD8+ T cells, but metronidazole can decrease Fusobacterium nucleatum and improve response to anti-PD-1 mAb	
Nanotechnology	Nanoparticles can target Asporin to improve oxaliplatin sensitivity Nanoparticles can co-deliver 5-FU and pro-apoptotic factor L3 to overcome chemoresistance	Cetuximab-conjugated gold nanoparticles can increase EGFR endocytosis and degradation to decrease tumor growth.	Nanoparticles can silence YTHDF1 to re-sensitize cells to anti-PD-1	Albumin-coated MnO2 nanoparticles can relieve hypoxia in the tumor microenvironment to overcome radiotherapy resistance.

Nanotechnology has emerged as a novel approach to overcoming CRC resistance by enabling precise drug delivery and enhancing therapeutic efficacy. Nanoparticles, defined as particles measuring 1 to 100 nanometers that have specialized surface properties, have been successfully used for chemotherapy and adjunctive gene therapy (169). Various methods of nanoparticle delivery have emerged over the years, including co-delivery of nanoparticles for synergistic effects as well as conjugation of nanoparticles with treatments, proteins, and charged particles for enhanced therapeutic effect. For example, elevated Asporin (ASPN) expression in oxaliplatin-resistant CRC was targeted using nanoparticles co-delivering ASPN-siRNA and oxaliplatin, resulting in improved anti-tumor activity *in vitro* and *in vivo* (170). Another strategy utilized NPs co-delivering 5-FU and the pro-apoptotic factor L3 to regulate p-gp expression and overcome chemoresistance (171). Liposomes formulations of irinotecan and curcumin also demonstrated a synergistic effect in CRC, with curcumin enhancing activation of irinotecan and increased expression of the targets of the active metabolite SN-38 (172). To overcome radiotherapy resistance due to a hypoxic tumor environment, albumin-coated MnO2 nanoparticles have been developed to relieve hypoxia in the TME (173). Resistance of cetuximab was reversed in CRC from administering cetuximab-conjugated gold NPs, which increased EGFR endocytosis and degradation, leading to suppression of downstream signaling pathways and decreased tumor growth (174). In terms of immunotherapy for CRC, Ni et al. created a bi-adjuvant neoantigen nanovaccine (banNV) within a nanoparticle that consisted of peptide neoantigen Adpgk and adjuvants Toll-like receptor (TLR) 7/8 agonist R848 and TLR9 agonist CpG. When administered with anti-PD-1, significant regression of CRC tumors was found (175). Immune-modifying, negatively charged nanoparticles ONP-302 were shown to inhibit CRC tumor growth and suppress pro-tumorigenic gene expression in TAFs (176). While the nanotechnology is a potent field for cancer therapeutics, there have been concerns for toxicity in dosing and the propensity to incite inflammation and worsen respiratory and cardiovascular disease. Therefore, careful design and administration are warranted to minimize adverse effects with nanoparticle-related treatments (177).

Although significant progress has been made in advancing the use of nanotechnology in treating cancer, further optimization of nanoparticle therapy targeting TAFs in CRC specifically is warranted (178). Previously, patient-derived xenografts and patient-derived organoids have been used as pre-clinical models of CRC. However, for use in making clinical decisions for patients, these models are too inefficient and slow. Therefore, there is a need for the development of new pre-clinical models that can be produced in a timely manner. Micro-organospheres have been found to be a promising new model of metastatic colorectal cancer in predicting which drugs can respond to a patient’s tumor within 14 days. Further investigation of this model with a greater number of patients is needed to confirm the use of this model in future CRC experiments and patients (179).

Based on our research and synthesis of the literature, we propose a translational roadmap for guiding treatment decisions in colorectal cancer (CRC) by integrating biomarker screening with mechanistic understanding of drug resistance. Clinically, molecular profiling of *KRAS*, *BRAF*, *NRAS*, *PIK3CA*, *TP53*, and *MMR/MSI* status remains foundational for stratifying patients for targeted therapies and immunotherapies. However, our recent findings underscore the critical role of the tumor microenvironment, particularly neutrophil extracellular traps (NETs), in promoting immune evasion, and metastasis (180–183). We have demonstrated that NETs drive CRC liver metastasis by remodeling the immune landscape and impeding CD8^+^ T cell function, and that DNase I-mediated degradation of NETs can reverse this resistance and restore antitumor immunity (181, 182). Therefore, we propose incorporating NET biomarkers (e.g., MPO-DNA complexes, citrullinated histones) into liquid biopsy panels for high-risk or refractory CRC cases. In parallel, profiling immune cell composition—such as tumor-infiltrating Tregs, MDSCs, and macrophage polarization states—may help determine the appropriateness of immunomodulatory strategies. For patients exhibiting resistance to conventional therapy, we advocate combination regimens that include NET-targeting agents (e.g., DNase I, PAD4 inhibitors), ferroptosis inducers, or nanoparticle-based drug delivery systems that overcome stromal barriers. This roadmap, while still in need of prospective clinical validation, offers a mechanism-based approach to personalize therapy and counteract resistance in CRC.

## 8 Discussion

Drug resistance remains a major challenge in the effective treatment of CRC, contributing to its poor prognosis and high recurrence rates. In this review, we provided a comprehensive overview of the mechanisms of resistance in CRC, categorized by therapy type. Chemotherapy resistance mechanisms were linked to evasion of apoptosis and ferroptosis, increased drug inactivation, decreased drug activation, changes in drug transport, modulations in drug targets, and interactions with components of the TME, such as cancer-associated fibroblasts and tumor-associated macrophages. Resistance to targeted therapies was attributed to alterations in drug targets, aberrant receptor activation, modulation to signaling and angiogenesis pathways, the involvement of cancer stem cells, and metabolic reprogramming. Immunotherapy resistance in CRC included mechanisms such as mutations of HLA complexes as well as involvement of cancer-associated fibroblasts and myeloid-derived suppressor cells in the TME. Resistance to radiotherapy was associated with the establishment of hypoxic tumor microenvironments and other adaptive responses.

This review also highlighted shared resistance mechanisms across treatment modalities and discussed emerging strategies to overcome these barriers. Among these, nanotechnology-based approaches hold significant promise for enhancing therapeutic efficacy by circumventing resistance mechanisms. Although further investigation and rigorous preclinical and clinical evaluations are essential to fully develop these emerging therapeutics, the expanding understanding of CRC resistance mechanisms and advancements in innovative treatment strategies provide a hopeful outlook for improving CRC management and patient outcomes.

Looking forward, the clinical translation of these emerging strategies will require overcoming several technical and biological hurdles. For instance, while nanoparticle-based drug delivery can improve tumor specificity and reduce systemic toxicity, challenges such as limited penetration into hypoxic tumor cores and variability in patient response remain. Similarly, the application of personalized medicine—guided by genomic and transcriptomic profiling—relies on reliable biomarkers, which are still under development for many resistance mechanisms. Overcoming these barriers will require integrated efforts, including the refinement of preclinical models that faithfully recapitulate human CRC resistance, advances in real-time imaging and biosensing, and the implementation of adaptive clinical trial designs. Collaborative, multidisciplinary research will be essential to bridge the gap between experimental innovation and clinical application, ultimately paving the way for more effective, individualized CRC therapies.

## References

[1] Colorectal Cancer Alliance. ACS releases colorectal cancer estimates for 2024. Washington, DC: Colorectal Cancer Alliance (2024). Available online at: https://colorectalcancer.org/article/acs-releases-colorectal-cancer-estimates-2024.

[2] Xie YH Chen YX Fang JY. Comprehensive review of targeted therapy for colorectal cancer. Signal Transduct Target Ther. (2020) 5:1–30. doi: 10.1038/s41392-020-0116-z, PMID: 32296018 PMC7082344

[3] American Cancer Society. Radiation Therapy for Colorectal Cancer | Radiation for Colorectal Cancer . Available online at: https://www.cancer.org/cancer/types/colon-rectal-cancer/treating/radiation-therapy.html (Accessed July 23, 2024).

[4] Nors J Iversen LH Erichsen R Gotschalck KA Andersen CL. Incidence of recurrence and time to recurrence in stage I to III colorectal cancer: A nationwide danish cohort study. JAMA Oncol. (2024) 10:54–62. doi: 10.1001/jamaoncol.2023.5098, PMID: 37971197 PMC10654928

[5] American Cancer Society. Cancer Facts & Figures 2024. Atlanta, GA (2024).

[6] CDC. Health and Economic Benefits of Colorectal Cancer Interventions. Atlanta, GA: National Center for Chronic Disease Prevention and Health Promotion (NCCDPHP (2024). Available online at: https://www.cdc.gov/nccdphp/priorities/colorectal-cancer.html.

[7] Blondy S David V Verdier M Mathonnet M Perraud A Christou N. 5-Fluorouracil resistance mechanisms in colorectal cancer: From classical pathways to promising processes. Cancer Sci. (2020) 111:3142–54. doi: 10.1111/cas.14532, PMID: 32536012 PMC7469786

[8] Bukowski K Kciuk M Kontek R. Mechanisms of multidrug resistance in cancer chemotherapy. Int J Mol Sci. (2020) 21:3233. doi: 10.3390/ijms21093233, PMID: 32370233 PMC7247559

[9] Lei Z Tian Q Teng Q Wurpel JND Zeng L Pan Y. Understanding and targeting resistance mechanisms in cancer. MedComm. (2023) 4:e265. doi: 10.1002/mco2.265, PMID: 37229486 PMC10203373

[10] Khan SU Fatima K Aisha S Malik F. Unveiling the mechanisms and challenges of cancer drug resistance. Cell Commun Signal. (2024) 22:109. doi: 10.1186/s12964-023-01302-1, PMID: 38347575 PMC10860306

[11] Danial NN Korsmeyer SJ. Cell death: critical control points. Cell. (2004) 116:205–19. doi: 10.1016/S0092-8674(04)00046-7, PMID: 14744432

[12] Fulda S. Tumor resistance to apoptosis. Int J Cancer. (2009) 124:511–5. doi: 10.1002/ijc.24064, PMID: 19003982

[13] Pfeffer CM Singh ATK. Apoptosis: A target for anticancer therapy. Int J Mol Sci. (2018) 19:448. doi: 10.3390/ijms19020448, PMID: 29393886 PMC5855670

[14] Zhu G Pan C Bei JX Li B Liang C Xu Y. Mutant p53 in cancer progression and targeted therapies. Front Oncol. (2020) 10:595187. doi: 10.3389/fonc.2020.595187, PMID: 33240819 PMC7677253

[15] Liebl MC Hofmann TG. The role of p53 signaling in colorectal cancer. Cancers. (2021) 13:2125. doi: 10.3390/cancers13092125, PMID: 33924934 PMC8125348

[16] Boyer J McLean EG Aroori S Wilson P McCulla A Carey PD. Characterization of p53 wild-type and null isogenic colorectal cancer cell lines resistant to 5-fluorouracil, oxaliplatin, and irinotecan. Clin Cancer Res. (2004) 10:2158–67. doi: 10.1158/1078-0432.CCR-03-0362, PMID: 15041737

[17] Yao J Huang A Zheng X Liu T Lin Z Zhang S. 53BP1 loss induces chemoresistance of colorectal cancer cells to 5-fluorouracil by inhibiting the ATM-CHK2-P53 pathway. J Cancer Res Clin Oncol. (2017) 143:419–31. doi: 10.1007/s00432-016-2302-5, PMID: 27838786 PMC11819077

[18] Kandioler D Mittlböck M Kappel S Puhalla H Herbst F Langner C. TP53 mutational status and prediction of benefit from adjuvant 5-fluorouracil in stage III colon cancer patients. EBioMedicine. (2015) 2:825–30. doi: 10.1016/j.ebiom.2015.06.003, PMID: 26425688 PMC4563117

[19] Abbas R Larisch S. Targeting XIAP for promoting cancer cell death—The story of ARTS and SMAC. Cells. (2020) 9:663. doi: 10.3390/cells9030663, PMID: 32182843 PMC7140716

[20] Xiong Z Fu Z Shi J Jiang X Wan H. HtrA1 down-regulation induces cisplatin resistance in colon cancer by increasing XIAP and activating PI3K/akt pathway. Ann Clin Lab Sci. (2017) 47:264–70. doi: 10.1016/j.biopha.2021.111603, PMID: 28667026

[21] Zhang Y Talmon G Wang J. MicroRNA-587 antagonizes 5-FU-induced apoptosis and confers drug resistance by regulating PPP2R1B expression in colorectal cancer. Cell Death Dis. (2015) 6:e1845. doi: 10.1038/cddis.2015.200, PMID: 26247730 PMC4558495

[22] Zhang L Yu J Park BH Kinzler KW Vogelstein B. Role of BAX in the apoptotic response to anticancer agents. Science. (2000) 290:989–92. doi: 10.1126/science.290.5493.989, PMID: 11062132

[23] Timme CR Gruidl M Yeatman TJ. Gamma-secretase inhibition attenuates oxaliplatin-induced apoptosis through increased Mcl-1 and/or Bcl-xL in human colon cancer cells. Apoptosis. (2013) 18:1163–74. doi: 10.1007/s10495-013-0883-x, PMID: 23887890

[24] Fan H Zhu JH Yao XQ. Knockdown of long non-coding RNA PVT1 reverses multidrug resistance in colorectal cancer cells. Mol Med Rep. (2018) 17:8309–15. doi: 10.3892/mmr.2018.8907, PMID: 29693171 PMC5984006

[25] Chaudhary N Choudhary BS Shah SG Khapare N Dwivedi N Gaikwad A. Lipocalin 2 expression promotes tumor progression and therapy resistance by inhibiting ferroptosis in colorectal cancer. Int J Cancer. (2021) 149:1495–511. doi: 10.1002/ijc.33711, PMID: 34146401

[26] Yang C Zhang Y Lin S Liu Y Li W. Suppressing the KIF20A/NUAK1/Nrf2/GPX4 signaling pathway induces ferroptosis and enhances the sensitivity of colorectal cancer to oxaliplatin. Aging. (2021) 13:13515–34. doi: 10.18632/aging.202774, PMID: 33819186 PMC8202845

[27] Mou Y Wang J Wu J He D Zhang C Duan C. Ferroptosis, a new form of cell death: opportunities and challenges in cancer. J Hematol OncolJ Hematol Oncol. (2019) 12:34. doi: 10.1186/s13045-019-0720-y, PMID: 30925886 PMC6441206

[28] Kang KA Piao MJ Kim KC Kang HK Chang WY Park IC. Epigenetic modification of Nrf2 in 5-fluorouracil-resistant colon cancer cells: involvement of TET-dependent DNA demethylation. Cell Death Dis. (2014) 5:e1183. doi: 10.1038/cddis.2014.149, PMID: 24743738 PMC4001304

[29] van Kuilenburg ABP. Dihydropyrimidine dehydrogenase and the efficacy and toxicity of 5-fluorouracil. Eur J Cancer. (2004) 40:939–50. doi: 10.1016/j.ejca.2003.12.004, PMID: 15093568

[30] Soong R Shah N Salto-Tellez M Tai BC Soo RA Han HC. Prognostic significance of thymidylate synthase, dihydropyrimidine dehydrogenase and thymidine phosphorylase protein expression in colorectal cancer patients treated with or without 5-fluorouracil-based chemotherapy. Ann Oncol Off J Eur Soc Med Oncol ESMO. (2008) 19:915–9. doi: 10.1093/annonc/mdm599, PMID: 18245778 PMC2931808

[31] Griffith M Mwenifumbo JC Cheung PY Paul JE Pugh TJ Tang MJ. Novel mRNA isoforms and mutations of uridine monophosphate synthetase and 5-fluorouracil resistance in colorectal cancer. Pharmacogenom J. (2013) 13:148–58. doi: 10.1038/tpj.2011.65, PMID: 22249354

[32] Humeniuk R Menon LG Mishra PJ Gorlick R Sowers R Rode W. Decreased levels of UMP kinase as a mechanism of fluoropyrimidine resistance. Mol Cancer Ther. (2009) 8:1037–44. doi: 10.1158/1535-7163.MCT-08-0716, PMID: 19383847

[33] Lu LL Chen XH Zhang G Liu Z-C Wu N Wang H. CCL21 facilitates chemoresistance and cancer stem cell-like properties of colorectal cancer cells through AKT/GSK-3β/snail signals. Oxid Med Cell Longev. (2016) 2016:5874127. doi: 10.1155/2016/5874127, PMID: 27057280 PMC4707330

[34] Meschini S Calcabrini A Monti E Del Bufalo D Stringaro A Dolfini E. Intracellular P-glycoprotein expression is associated with the intrinsic multidrug resistance phenotype in human colon adenocarcinoma cells. Int J Cancer. (2000) 87:615–28. doi: 10.1002/1097-0215(20000901)87:5<615::AID-IJC1>3.0.CO;2-4, PMID: 10925353

[35] Spoelstra EC Dekker H Schuurhuis GJ Broxterman HJ Lankelma J. P-glycoprotein drug efflux pump involved in the mechanisms of intrinsic drug resistance in various colon cancer cell lines: Evidence for a saturation of active daunorubicin transport. Biochem Pharmacol. (1991) 41:349–59. doi: 10.1016/0006-2952(91)90531-9, PMID: 1671638

[36] Zhou H Lin C Zhang Y Zhang X Zhang C Zhang P. miR-506 enhances the sensitivity of human colorectal cancer cells to oxaliplatin by suppressing MDR1/P-gp expression. Cell Prolif. (2017) 50:e12341. doi: 10.1111/cpr.12341, PMID: 28217977 PMC6529089

[37] Wang T Chen Z Zhu Y Pan Q Liu Y Qi X. Inhibition of transient receptor potential channel 5 reverses 5-fluorouracil resistance in human colorectal cancer cells. J Biol Chem. (2015) 290:448–56. doi: 10.1074/jbc.M114.590364, PMID: 25404731 PMC4281746

[38] Du J He Y Li P Wu W Chen Y Ruan H. IL-8 regulates the doxorubicin resistance of colorectal cancer cells via modulation of multidrug resistance 1 (MDR1). Cancer Chemother Pharmacol. (2018) 81:1111–9. doi: 10.1007/s00280-018-3584-x, PMID: 29693201

[39] Herraez E Gonzalez-Sanchez E Vaquero J Romero MR Serrano MA Marin JJG. Cisplatin-induced chemoresistance in colon cancer cells involves FXR-dependent and FXR-independent up-regulation of ABC proteins. Mol Pharm. (2012) 9:2565–76. doi: 10.1021/mp300178a, PMID: 22800197

[40] Jung KA Choi BH Kwak MK. The c-MET/PI3K signaling is associated with cancer resistance to doxorubicin and photodynamic therapy by elevating BCRP/ABCG2 expression. Mol Pharmacol. (2015) 87:465–76. doi: 10.1124/mol.114.096065, PMID: 25534417

[41] Ross DD Yang W Abruzzo LV Dalton WS Schneider E Lage H. Atypical multidrug resistance: breast cancer resistance protein messenger RNA expression in mitoxantrone-selected cell lines. J Natl Cancer Inst. (1999) 91:429–33. doi: 10.1093/jnci/91.5.429, PMID: 10070941

[42] Zhu MM Tong JL Xu Q Nie F Xu XT Xiao SD. Increased JNK1 signaling pathway is responsible for ABCG2-mediated multidrug resistance in human colon cancer. PloS One. (2012) 7:e41763. doi: 10.1371/journal.pone.0041763, PMID: 22870247 PMC3411563

[43] Hinoshita E Uchiumi T Taguchi K Kinukawa N Tsuneyoshi M Maehara Y. Increased expression of an ATP-binding cassette superfamily transporter, multidrug resistance protein 2, in human colorectal carcinomas. Clin Cancer Res Off J Am Assoc Cancer Res. (2000) 6:2401–7., PMID: 10873092

[44] Liu Z Qiu M Tang QL Liu M Lang N Bi F. Establishment and biological characteristics of oxaliplatin-resistant human colon cancer cell lines. Chin J Cancer. (2010) 29:661–7. doi: 10.5732/cjc.009.10666, PMID: 20591218

[45] Wilson BJ Schatton T Zhan Q Gasser M Ma J Saab KR. ABCB5 identifies a therapy-refractory tumor cell population in colorectal cancer patients. Cancer Res. (2011) 71:5307–16. doi: 10.1158/0008-5472.CAN-11-0221, PMID: 21652540 PMC3395026

[46] Liu X Wu B Chen H Sun H Guo X Sun T. Intense endoplasmic reticulum stress (ERS)/IRE1α enhanced Oxaliplatin efficacy by decreased ABCC10 in colorectal cancer cells. BMC Cancer. (2022) 22:1369. doi: 10.1186/s12885-022-10415-8, PMID: 36585626 PMC9805014

[47] Xie T Geng J Wang Y Wang L Huang M Chen J. FOXM1 evokes 5-fluorouracil resistance in colorectal cancer depending on ABCC10. Oncotarget. (2016) 8:8574–89. doi: 10.18632/oncotarget.14351, PMID: 28051999 PMC5352423

[48] Kang YH Lee JS Lee NH Kim SH Seo CS Son CG. Coptidis rhizoma extract reverses 5-fluorouracil resistance in HCT116 human colorectal cancer cells via modulation of thymidylate synthase. Mol Basel Switz. (2021) 26:1856. doi: 10.3390/molecules26071856, PMID: 33806077 PMC8036817

[49] Varghese V Magnani L Harada-Shoji N Mauri F Szydlo RM Yao S. FOXM1 modulates 5-FU resistance in colorectal cancer through regulating TYMS expression. Sci Rep. (2019) 9:1505. doi: 10.1038/s41598-018-38017-0, PMID: 30728402 PMC6365533

[50] Ahn JY Lee JS Min HY Lee HY. Acquired resistance to 5-fluorouracil via HSP90/Src-mediated increase in thymidylate synthase expression in colon cancer. Oncotarget. (2015) 6:32622–33. doi: 10.18632/oncotarget.5327, PMID: 26416450 PMC4741717

[51] Liu R Li J Xie K Zhang T Lei Y Chen Y. FGFR4 promotes stroma-induced epithelial-to-mesenchymal transition in colorectal cancer. Cancer Res. (2013) 73:5926–35. doi: 10.1158/0008-5472.CAN-12-4718, PMID: 23943801

[52] Turkington RC Longley DB Allen WL Stevenson L McLaughlin K Dunne PD. Fibroblast growth factor receptor 4 (FGFR4): a targetable regulator of drug resistance in colorectal cancer. Cell Death Dis. (2014) 5:e1046. doi: 10.1038/cddis.2014.10, PMID: 24503538 PMC3944229

[53] Guillén Díaz-Maroto N Sanz-Pamplona R Berdiel-Acer M Cimas FJ García E Gonçalves-Ribeiro S. Noncanonical TGFβ Pathway relieves the blockade of IL1β/TGFβ-mediated crosstalk between tumor and stroma: TGFBR1 and TAK1 inhibition in colorectal cancer. Clin Cancer Res. (2019) 25:4466–79. doi: 10.1158/1078-0432.CCR-18-3957, PMID: 30979739

[54] Tang YA Chen Yf Bao Y Mahara S Yatim SMJM Oguz G. Hypoxic tumor microenvironment activates GLI2 via HIF-1α and TGF-β2 to promote chemoresistance in colorectal cancer. Proc Natl Acad Sci U S A. (2018) 115:E5990–9. doi: 10.1073/pnas.1801348115, PMID: 29891662 PMC6042102

[55] Li Z Chan K Qi Y Lu L Ning F Wu M. Participation of CCL1 in snail-positive fibroblasts in colorectal cancer contribute to 5-fluorouracil/paclitaxel chemoresistance. Cancer Res Treat Off J Korean Cancer Assoc. (2018) 50:894–907. doi: 10.4143/crt.2017.356, PMID: 28934847 PMC6056976

[56] Zhang X Chen Y Hao L Hou A Chen X Li Y. Macrophages induce resistance to 5-fluorouracil chemotherapy in colorectal cancer through the release of putrescine. Cancer Lett. (2016) 381:305–13. doi: 10.1016/j.canlet.2016.08.004, PMID: 27514455

[57] Zhang L Lu X Xu Y La X Tian J Li A. Tumor-associated macrophages confer colorectal cancer 5-fluorouracil resistance by promoting MRP1 membrane translocation via an intercellular CXCL17/CXCL22-CCR4-ATF6-GRP78 axis. Cell Death Dis. (2023) 14:582. doi: 10.1038/s41419-023-06108-0, PMID: 37658050 PMC10474093

[58] Liao W Shi M Zeng D Huang N Wang Y Li J. Tumor and microenvironment evolution during chemotherapy combine with bevacizumab in colorectal cancer liver metastasis. J Clin Oncol. (2019) 37:3568–8. doi: 10.1200/JCO.2019.37.15_suppl.3568

[59] Yin Y Yao S Hu Y Feng Y Li M Bian Z. The immune-microenvironment confers chemoresistance of colorectal cancer through macrophage-derived IL6. Clin Cancer Res Off J Am Assoc Cancer Res. (2017) 23:7375–87. doi: 10.1158/1078-0432.CCR-17-1283, PMID: 28928161

[60] Luo J Wang C Su J Yi T Tang S. CUL4B increases platinum-based drug resistance in colorectal cancer through EMT: A study in its mechanism. J Cell Mol Med. (2022) 26:5767–78. doi: 10.1111/jcmm.17585, PMID: 36385733 PMC9716322

[61] Zhang J Miller Z Musich PR Thomas AE Yao ZQ Xie Q. DSTYK promotes metastasis and chemoresistance via EMT in colorectal cancer. Front Pharmacol. (2020) 11:1250. doi: 10.3389/fphar.2020.01250, PMID: 32982725 PMC7493073

[62] Zhu Y Huang S Chen S Chen J Wang Z Wang Y. SOX2 promotes chemoresistance, cancer stem cells properties, and epithelial–mesenchymal transition by β-catenin and Beclin1/autophagy signaling in colorectal cancer. Cell Death Dis. (2021) 12:449. doi: 10.1038/s41419-021-03733-5, PMID: 33953166 PMC8100126

[63] Hu YB Yan C Mu L Mi Y-L Zhao H Hu H. Exosomal Wnt-induced dedifferentiation of colorectal cancer cells contributes to chemotherapy resistance. Oncogene. (2019) 38:1951–65. doi: 10.1038/s41388-018-0557-9, PMID: 30390075 PMC6756234

[64] Wang X Zhang H Yang H Bai M Ning T Deng T. Exosome-delivered circRNA promotes glycolysis to induce chemoresistance through the miR-122-PKM2 axis in colorectal cancer. Mol Oncol. (2020) 14:539–55. doi: 10.1002/1878-0261.12629, PMID: 31901148 PMC7053238

[65] Arena S Bellosillo B Siravegna G Martínez A Cañadas I Lazzari L. Emergence of multiple EGFR extracellular mutations during cetuximab treatment in colorectal cancer. Clin Cancer Res Off J Am Assoc Cancer Res. (2015) 21:2157–66. doi: 10.1158/1078-0432.CCR-14-2821, PMID: 25623215

[66] Liao HW Hsu JM Xia W Wang H-L Wang Y-N Chang W-C. PRMT1-mediated methylation of the EGF receptor regulates signaling and cetuximab response. J Clin Invest. (2015) 125:4529–43. doi: 10.1172/JCI82826, PMID: 26571401 PMC4665782

[67] Montagut C Dalmases A Bellosillo B Crespo M Pairet S Iglesias M. Identification of a mutation in the extracellular domain of the Epidermal Growth Factor Receptor conferring cetuximab resistance in colorectal cancer. Nat Med. (2012) 18:221–3. doi: 10.1038/nm.2609, PMID: 22270724

[68] Jacobs B De Roock W Piessevaux H Van Oirbeek R Biesmans B De Schutter J. Amphiregulin and epiregulin mRNA expression in primary tumors predicts outcome in metastatic colorectal cancer treated with cetuximab. J Clin Oncol. (2009) 27:5068–74. doi: 10.1200/JCO.2008.21.3744, PMID: 19738126

[69] Khambata-Ford S Garrett CR Meropol NJ Basik M Harbison CT Wu S. Expression of epiregulin and amphiregulin and K-ras mutation status predict disease control in metastatic colorectal cancer patients treated with cetuximab. J Clin Oncol. (2007) 25:3230–7. doi: 10.1200/JCO.2006.10.5437, PMID: 17664471

[70] Wang Q Shen X Chen G Du J. Drug resistance in colorectal cancer: from mechanism to clinic. Cancers. (2022) 14:2928. doi: 10.3390/cancers14122928, PMID: 35740594 PMC9221177

[71] Scartozzi M Giampieri R Maccaroni E Mandolesi A Giustini L Silva R. Analysis of HER-3, insulin growth factor-1, nuclear factor-kB and epidermal growth factor receptor gene copy number in the prediction of clinical outcome for K-RAS wild-type colorectal cancer patients receiving irinotecan–cetuximab. Ann Oncol. (2012) 23:1706–12. doi: 10.1093/annonc/mdr558, PMID: 22112971

[72] Zanella ER Galimi F Sassi F Migliardi G Cottino F Leto SM. IGF2 is an actionable target that identifies a distinct subpopulation of colorectal cancer patients with marginal response to anti-EGFR therapies. Sci Transl Med. (2015) 7:272ra12–272ra12. doi: 10.1126/scitranslmed.3010445, PMID: 25632036

[73] Han Y Peng Y Fu Y Cai C Guo C Liu S. MLH1 deficiency induces cetuximab resistance in colon cancer via her-2/PI3K/AKT signaling. Adv Sci. (2020) 7:2000112. doi: 10.1002/advs.202000112, PMID: 32670759 PMC7341094

[74] Bertotti A Migliardi G Galimi F Cai C Guo C Liu S. A molecularly annotated platform of patient-derived xenografts (“xenopatients”) identifies HER2 as an effective therapeutic target in cetuximab-resistant colorectal cancer. Cancer Discov. (2011) 1:508–23. doi: 10.1158/2159-8290.CD-11-0109, PMID: 22586653

[75] Kavuri SM Jain N Galimi F Cottino F Leto SM Migliardi G. HER2 activating mutations are targets for colorectal cancer treatment. Cancer Discov. (2015) 5:832–41. doi: 10.1158/2159-8290.CD-14-1211, PMID: 26243863 PMC4527087

[76] Yonesaka K Zejnullahu K Okamoto I Satoh T Cappuzzo F Souglakos J. Activation of ERBB2 signaling causes resistance to the EGFR-directed therapeutic antibody cetuximab. Sci Transl Med. (2011) 3:99ra86. doi: 10.1126/scitranslmed.3002442, PMID: 21900593 PMC3268675

[77] Bardelli A Corso S Bertotti A Hobor S Valtorta E Siravegna G. Amplification of the MET receptor drives resistance to anti-EGFR therapies in colorectal cancer. Cancer Discov. (2013) 3:658–73. doi: 10.1158/2159-8290.CD-12-0558, PMID: 23729478 PMC4078408

[78] Amado RG Wolf M Peeters M Van Cutsem E Siena S Freeman DJ. Wild-type KRAS is required for panitumumab efficacy in patients with metastatic colorectal cancer. J Clin Oncol. (2008) 26:1626–34. doi: 10.1200/JCO.2007.14.7116, PMID: 18316791

[79] Luraghi P Reato G Cipriano E Sassi F Orzan F Bigatto V. MET signaling in colon cancer stem-like cells blunts the therapeutic response to EGFR inhibitors. Cancer Res. (2014) 74:1857–69. doi: 10.1158/0008-5472.CAN-13-2340-T, PMID: 24448239

[80] Woolston A Khan K Spain G Barber LJ Griffiths B Gonzalez-Exposito R. Genomic and transcriptomic determinants of therapy resistance and immune landscape evolution during anti-EGFR treatment in colorectal cancer. Cancer Cell. (2019) 36:35–50.e9. doi: 10.1016/j.ccell.2019.05.013, PMID: 31287991 PMC6617392

[81] Lin SP Lee YT Yang SH Miller SA Chiou S-H Hung M-C. Colon cancer stem cells resist antiangiogenesis therapy-induced apoptosis. Cancer Lett. (2013) 328:226–34. doi: 10.1016/j.canlet.2012.08.036, PMID: 23017941

[82] Rahbari NN Kedrin D Incio J Liu H Ho WW Nia HT. Anti-VEGF therapy induces ECM remodeling and mechanical barriers to therapy in colorectal cancer liver metastases. Sci Transl Med. (2016) 8:360ra135. doi: 10.1126/scitranslmed.aaf5219, PMID: 27733559 PMC5457741

[83] Vallböhmer D Zhang W Gordon M Yang DY Yun J Press OA. Molecular determinants of cetuximab efficacy. J Clin Oncol Off J Am Soc Clin Oncol. (2005) 23:3536–44. doi: 10.1200/JCO.2005.09.100, PMID: 15908664

[84] Kopetz S Hoff PM Morris JS Wolff RA Eng C Glover KY. Phase II trial of infusional fluorouracil, irinotecan, and bevacizumab for metastatic colorectal cancer: efficacy and circulating angiogenic biomarkers associated with therapeutic resistance. J Clin Oncol. (2010) 28:453–9. doi: 10.1200/JCO.2009.24.8252, PMID: 20008624 PMC2815707

[85] Goede V Coutelle O Neuneier J Reinacher-Schick A Schnell R Koslowsky TC. Identification of serum angiopoietin-2 as a biomarker for clinical outcome of colorectal cancer patients treated with bevacizumab-containing therapy. Br J Cancer. (2010) 103:1407–14. doi: 10.1038/sj.bjc.6605925, PMID: 20924372 PMC2990609

[86] Maddalena F Condelli V Matassa DS Pacelli C Scrima R Lettini G. TRAP1 enhances Warburg metabolism through modulation of PFK1 expression/activity and favors resistance to EGFR inhibitors in human colorectal carcinomas. Mol Oncol. (2020) 14:3030–47. doi: 10.1002/1878-0261.12814, PMID: 33025742 PMC7718945

[87] Zheng Y Zhou R Cai J Yang N Wen Z Zhang Z. Matrix stiffness triggers lipid metabolic cross-talk between tumor and stromal cells to mediate bevacizumab resistance in colorectal cancer liver metastases. Cancer Res. (2023) 83:3577–92. doi: 10.1158/0008-5472.CAN-23-0025, PMID: 37610655 PMC10618741

[88] Skvortsov S Sarg B Loeffler-Ragg J Skvortsova I Lindner H Ott H Werner. Different proteome pattern of epidermal growth factor receptor-positive colorectal cancer cell lines that are responsive and nonresponsive to C225 antibody treatment. Mol Cancer Ther. (2004) 3:1551–8. doi: 10.1158/1535-7163.1551.3.12, PMID: 15634648

[89] Carbone C Piro G Simionato F Ligorio F Cremolini C Loupakis F. Homeobox B9 mediates resistance to anti-VEGF therapy in colorectal cancer patients. Clin Cancer Res Off J Am Assoc Cancer Res. (2017) 23:4312–22. doi: 10.1158/1078-0432.CCR-16-3153, PMID: 28298545

[90] Johnson RM Qu X Lin CF Huw L-Y Venkatanarayan A Sokol E. ARID1A mutations confer intrinsic and acquired resistance to cetuximab treatment in colorectal cancer. Nat Commun. (2022) 13:5478. doi: 10.1038/s41467-022-33172-5, PMID: 36117191 PMC9482920

[91] Le DT Durham JN Smith KN Wang H Bartlett BR Aulakh LK. Mismatch-repair deficiency predicts response of solid tumors to PD-1 blockade. Science. (2017) 357:409–13. doi: 10.1126/science.aan6733, PMID: 28596308 PMC5576142

[92] Grasso CS Giannakis M Wells DK Hamada T Mu XJ Quist M. Genetic mechanisms of immune evasion in colorectal cancer. Cancer Discov. (2018) 8:730–49. doi: 10.1158/2159-8290.CD-17-1327, PMID: 29510987 PMC5984687

[93] Ozcan M Janikovits J von Knebel Doeberitz M Kloor M. Complex pattern of immune evasion in MSI colorectal cancer. Oncoimmunology. (2018) 7:e1445453. doi: 10.1080/2162402X.2018.1445453, PMID: 29900056 PMC5993484

[94] Tauriello DVF Palomo-Ponce S Stork D Berenguer-Llergo A Badia-Ramentol J Iglesias M. TGFβ drives immune evasion in genetically reconstituted colon cancer metastasis. Nature. (2018) 554:538–43. doi: 10.1038/nature25492, PMID: 29443964

[95] Hanley CJ Mellone M Ford K Thirdborough SM Mellows T Frampton SJ. Targeting the myofibroblastic cancer-associated fibroblast phenotype through inhibition of NOX4. JNCI J Natl Cancer Inst. (2017) 110:109–20. doi: 10.1093/jnci/djx121, PMID: 28922779 PMC5903651

[96] Chun E Lavoie S Michaud M Gallini CA Kim J Soucy G. CCL2 promotes colorectal carcinogenesis by enhancing polymorphonuclear myeloid-derived suppressor cell population and function. Cell Rep. (2015) 12:244–57. doi: 10.1016/j.celrep.2015.06.024, PMID: 26146082 PMC4620029

[97] Katoh H Wang D Daikoku T Sun H Dey SK DuBois RN. CXCR2-expressing myeloid-derived suppressor cells are essential to promote colitis-associated tumorigenesis. Cancer Cell. (2013) 24:631–44. doi: 10.1016/j.ccr.2013.10.009, PMID: 24229710 PMC3928012

[98] Wu P Wu D Ni C Ye J Chen W Hu G. γδT17 cells promote the accumulation and expansion of myeloid-derived suppressor cells in human colorectal cancer. Immunity. (2014) 40:785–800. doi: 10.1016/j.immuni.2014.03.013, PMID: 24816404 PMC4716654

[99] Yang R Cai T Wu X Liu Y He J Zhang X. Tumour YAP1 and PTEN expression correlates with tumour-associated myeloid suppressor cell expansion and reduced survival in colorectal cancer. Immunology. (2018) 155:263–72. doi: 10.1111/imm.12949, PMID: 29770434 PMC6142285

[100] Garton AJ Seibel S Lopresti-Morrow L Crew L Janson N Mandiyan S. Anti-KIT monoclonal antibody treatment enhances the antitumor activity of immune checkpoint inhibitors by reversing tumor-induced immunosuppression. Mol Cancer Ther. (2017) 16:671–80. doi: 10.1158/1535-7163.MCT-16-0676, PMID: 28138031

[101] Liao W Overman MJ Boutin AT Shang X Zhao D Dey P. KRAS-IRF2 axis drives immune suppression and immune therapy resistance in colorectal cancer. Cancer Cell. (2019) 35:559–572.e7. doi: 10.1016/j.ccell.2019.02.008, PMID: 30905761 PMC6467776

[102] Yuan J Li J Gao C Jiang C Xiang Z Wu J. Immunotherapies catering to the unmet medical need of cold colorectal cancer. Front Immunol. (2022) 13:1022190. doi: 10.3389/fimmu.2022.1022190, PMID: 36275766 PMC9579278

[103] Goc J Lv M Bessman NJ Flamar A-L Sahota S Suzuki H. Dysregulation of ILC3s unleashes progression and immunotherapy resistance in colon cancer. Cell. (2021) 184:5015–5030.e16. doi: 10.1016/j.cell.2021.07.029, PMID: 34407392 PMC8454863

[104] Tessmann JW Rocha MR Morgado-Díaz JA. Mechanisms of radioresistance and the underlying signaling pathways in colorectal cancer cells. J Cell Biochem. (2023) 124:31–45. doi: 10.1002/jcb.30361, PMID: 36565460

[105] Batlle E Clevers H. Cancer stem cells revisited. Nat Med. (2017) 23:1124–34. doi: 10.1038/nm.4409, PMID: 28985214

[106] Wu Y Song Y Wang R Wang T. Molecular mechanisms of tumor resistance to radiotherapy. Mol Cancer. (2023) 22:96. doi: 10.1186/s12943-023-01801-2, PMID: 37322433 PMC10268375

[107] Nicolas AM Pesic M Engel E Ziegler PK Diefenhardt M Kennel KB. Inflammatory fibroblasts mediate resistance to neoadjuvant therapy in rectal cancer. Cancer Cell. (2022) 40:168–184.e13. doi: 10.1016/j.ccell.2022.01.004, PMID: 35120600

[108] Wang H Jiang H Van De Gucht M De Ridder M. Hypoxic radioresistance: can ROS be the key to overcome it? Cancers. (2019) 11:112. doi: 10.3390/cancers11010112, PMID: 30669417 PMC6357097

[109] Wang X Lan Z He J Lai Q Yao X Li Q. LncRNA SNHG6 promotes chemoresistance through ULK1-induced autophagy by sponging miR-26a-5p in colorectal cancer cells. Cancer Cell Int. (2019) 19:234. doi: 10.1186/s12935-019-0951-6, PMID: 31516391 PMC6734319

[110] Zhang Y Li C Liu X Wang Y Zhao R Yang Y. circHIPK3 promotes oxaliplatin-resistance in colorectal cancer through autophagy by sponging miR-637. EBioMedicine. (2019) 48:277–88. doi: 10.1016/j.ebiom.2019.09.051, PMID: 31631038 PMC6838436

[111] Guo GF Jiang WQ Zhang B Cai Y-C Xu R-H Chen X-X. Autophagy-related proteins Beclin-1 and LC3 predict cetuximab efficacy in advanced colorectal cancer. World J Gastroenterol WJG. (2011) 17:4779. doi: 10.3748/wjg.v17.i43.4779, PMID: 22147978 PMC3229626

[112] Koustas E Sarantis P Theoharis S Saetta AA Chatziandreou I Kyriakopoulou G. Autophagy-related proteins as a prognostic factor of patients with colorectal cancer. Am J Clin Oncol. (2019) 42:767. doi: 10.1097/COC.0000000000000592, PMID: 31517637 PMC6766360

[113] Barr MP Gray SG Hoffmann AC Hilger RA Thomale J O’Flaherty JD. Generation and characterisation of cisplatin-resistant non-small cell lung cancer cell lines displaying a stem-like signature. PloS One. (2013) 8:e54193. doi: 10.1371/journal.pone.0054193, PMID: 23349823 PMC3547914

[114] Huynh N Shulkes A Baldwin G He H. Up-regulation of stem cell markers by P21-activated kinase 1 contributes to 5-fluorouracil resistance of colorectal cancer. Cancer Biol Ther. (2016) 17:813–23. doi: 10.1080/15384047.2016.1195045, PMID: 27260988 PMC5004687

[115] Ricci-Vitiani L Lombardi DG Pilozzi E Biffoni M Todaro M Peschle C. Identification and expansion of human colon-cancer-initiating cells. Nature. (2007) 445:111–5. doi: 10.1038/nature05384, PMID: 17122771

[116] Vermeulen L Todaro M Mello FdS Sprick MR Kemper K Alea MP. Single-cell cloning of colon cancer stem cells reveals a multi-lineage differentiation capacity. Proc Natl Acad Sci U S A. (2008) 105:13427. doi: 10.1073/pnas.0805706105, PMID: 18765800 PMC2533206

[117] Horst D Kriegl L Engel J Kirchner T Jung A. CD133 expression is an independent prognostic marker for low survival in colorectal cancer. Br J Cancer. (2008) 99:1285–9. doi: 10.1038/sj.bjc.6604664, PMID: 18781171 PMC2570510

[118] Liu G Yuan X Zeng Z Tunici P Ng H Abdulkadir IR. Analysis of gene expression and chemoresistance of CD133+ cancer stem cells in glioblastoma. Mol Cancer. (2006) 5:67. doi: 10.1186/1476-4598-5-67, PMID: 17140455 PMC1697823

[119] Smith LM Nesterova A Ryan MC Duniho S Jonas M Anderson M. CD133/prominin-1 is a potential therapeutic target for antibody-drug conjugates in hepatocellular and gastric cancers. Br J Cancer. (2008) 99:100–9. doi: 10.1038/sj.bjc.6604437, PMID: 18542072 PMC2453027

[120] Baskar R Dai J Wenlong N Yeo R Yeoh KW. Biological response of cancer cells to radiation treatment. Front Mol Biosci. (2014) 1. doi: 10.3389/fmolb.2014.00024, PMID: 25988165 PMC4429645

[121] Muzny DM Bainbridge MN Chang K Dinh HH Drummond JA Fowler G. Comprehensive molecular characterization of human colon and rectal cancer. Nature. (2012) 487:330–7. doi: 10.1038/nature11252, PMID: 22810696 PMC3401966

[122] Lee MA Park JH Rhyu SY Oh ST Kang WK Kim HN. Wnt3a expression is associated with MMP-9 expression in primary tumor and metastatic site in recurrent or stage IV colorectal cancer. BMC Cancer. (2014) 14:125. doi: 10.1186/1471-2407-14-125, PMID: 24564183 PMC3937452

[123] Chen S Guttridge DC You Z Zhang Z Fribley A Mayo MW. WNT-1 signaling inhibits apoptosis by activating β-catenin/T cell factor–mediated transcription. J Cell Biol. (2001) 152:87–96. doi: 10.1083/jcb.152.1.87, PMID: 11149923 PMC2193656

[124] Wu X Luo F Li J Zhong X Liu K. Tankyrase 1 inhibitior XAV939 increases chemosensitivity in colon cancer cell lines via inhibition of the Wnt signaling pathway. Int J Oncol. (2016) 48:1333–40. doi: 10.3892/ijo.2016.3360, PMID: 26820603 PMC4777596

[125] Almendro V Ametller E García-Recio S Collazo O Casas I Augé JM. The role of MMP7 and its cross-talk with the FAS/FASL system during the acquisition of chemoresistance to oxaliplatin. PloS One. (2009) 4:e4728. doi: 10.1371/journal.pone.0004728, PMID: 19266094 PMC2648894

[126] Li Q Yang T Li D Ding F Bai G Wang W. Knockdown of aquaporin-5 sensitizes colorectal cancer cells to 5-fluorouracil via inhibition of the Wnt–β-catenin signaling pathway. Biochem Cell Biol. (2018) 96:572–9. doi: 10.1139/bcb-2017-0162, PMID: 29390193

[127] Huang GL Song W Zhou P Fu Q-R Lin C-L Chen Q-X. Oncogenic retinoic acid receptor γ knockdown reverses multi-drug resistance of human colorectal cancer via Wnt/β-catenin pathway. Cell Cycle. (2017) 16:685–92. doi: 10.1080/15384101.2017.1295180, PMID: 28272990 PMC5397258

[128] Grainger S Nguyen N Richter J Setayesh J Lonquich B Oon CH. EGFR is required for Wnt9a–Fzd9b signalling specificity in haematopoietic stem cells. Nat Cell Biol. (2019) 21:721–30. doi: 10.1038/s41556-019-0330-5, PMID: 31110287 PMC6559346

[129] Lu Y Zhao X Liu Q Li C Graves-Deal R Cao Z. lncRNA MIR100HG-derived miR-100 and miR-125b mediate cetuximab resistance via Wnt/β-catenin signaling. Nat Med. (2017) 23:1331–41. doi: 10.1038/nm.4424, PMID: 29035371 PMC5961502

[130] Xiao Q Wu J Wang WJ Chen S Zheng Y Yu X. DKK2 imparts tumor immunity evasion through β-catenin-independent suppression of cytotoxic immune-cell activation. Nat Med. (2018) 24:262–70. doi: 10.1038/nm.4496, PMID: 29431745 PMC5840007

[131] Zhang H Bi Y Wei Y Liu J Kuerban K Ye L. Blocking wnt/β-catenin signal amplifies anti-PD-1 therapeutic efficacy by inhibiting tumor growth, migration, and promoting immune infiltration in glioblastomas. Mol Cancer Ther. (2021) 20:1305–15. doi: 10.1158/1535-7163.MCT-20-0825, PMID: 34001635

[132] Wang R Sun Q Wang P Liu M Xiong S Luo J. Notch and Wnt/β-catenin signaling pathway play important roles in activating liver cancer stem cells. Oncotarget. (2015) 7:5754–68. doi: 10.18632/oncotarget.6805, PMID: 26735577 PMC4868719

[133] Kumar V Vashishta M Kong L Wu X Lu JJ Guha C. The role of notch, hedgehog, and wnt signaling pathways in the resistance of tumors to anticancer therapies. Front Cell Dev Biol. (2021) 9:650772. doi: 10.3389/fcell.2021.650772, PMID: 33968932 PMC8100510

[134] Ma XT Wang S Ye YJ Du RY Cui ZR Somsouk M. Constitutive activation of Stat3 signaling pathway in human colorectal carcinoma. World J Gastroenterol. (2004) 10:1569–73. doi: 10.3748/wjg.v10.i11.1569, PMID: 15162527 PMC4572756

[135] Spitzner M Roesler B Bielfeld C Emons G Gaedcke J Wolff HA. STAT3 inhibition sensitizes colorectal cancer to chemoradiotherapy *in vitro* and *in vivo* . Int J Cancer. (2014) 134:997–1007. doi: 10.1002/ijc.28429, PMID: 23934972 PMC7706351

[136] Li Q Zhang D Chen X He L Li T Xu X. Nuclear PKM2 contributes to gefitinib resistance via upregulation of STAT3 activation in colorectal cancer. Sci Rep. (2015) 5:16082. doi: 10.1038/srep16082, PMID: 26542452 PMC4635355

[137] Dighe AS Richards E Old LJ Schreiber RD. Enhanced *in vivo* growth and resistance to rejection of tumor cells expressing dominant negative IFNγ receptors. Immunity. (1994) 1:447–56. doi: 10.1016/1074-7613(94)90087-6, PMID: 7895156

[138] Street SEA Cretney E Smyth MJ. Perforin and interferon-γ activities independently control tumor initiation, growth, and metastasis. Blood. (2001) 97:192–7. doi: 10.1182/blood.V97.1.192, PMID: 11133760

[139] Shin DS Zaretsky JM Escuin-Ordinas H Garcia-Diaz A Hu-Lieskovan S Kalbasi A. Primary resistance to PD-1 blockade mediated by JAK1/2 mutations. Cancer Discov. (2017) 7:188–201. doi: 10.1158/2159-8290.CD-16-1223, PMID: 27903500 PMC5296316

[140] Huang S Hölzel M Knijnenburg T Schlicker A Roepman P McDermott U. MED12 controls the response to multiple cancer drugs through regulation of TGF-β Receptor signaling. Cell. (2012) 151:937–50. doi: 10.1016/j.cell.2012.10.035, PMID: 23178117 PMC3672971

[141] Pirozzi G Tirino V Camerlingo R Franco R La Rocca A Liguori E. Epithelial to mesenchymal transition by TGFβ-1 induction increases stemness characteristics in primary non small cell lung cancer cell line. PloS One. (2011) 6:e21548. doi: 10.1371/journal.pone.0021548, PMID: 21738704 PMC3128060

[142] Romano G Santi L Bianco MR Giuffrè MR Pettinato M Bugarin C. The TGF-β pathway is activated by 5-fluorouracil treatment in drug resistant colorectal carcinoma cells. Oncotarget. (2016) 7:22077–91. doi: 10.18632/oncotarget.7895, PMID: 26956045 PMC5008345

[143] Quan Q Zhong F Wang X Chen K Guo L. PAR2 inhibition enhanced the sensitivity of colorectal cancer cells to 5-FU and reduced EMT signaling. Oncol Res. (2019) 27:779–88. doi: 10.3727/096504018X15442985680348, PMID: 30841957 PMC7848255

[144] Martin CJ Datta A Littlefield C Kalra A Chapron C Wawersik S. Selective inhibition of TGFβ1 activation overcomes primary resistance to checkpoint blockade therapy by altering tumor immune landscape. Sci Transl Med. (2020) 12:eaay8456. doi: 10.1126/scitranslmed.aay8456, PMID: 32213632

[145] Mariathasan S Turley SJ Nickles D Castiglioni A Yuen K Wang Y. TGFβ attenuates tumour response to PD-L1 blockade by contributing to exclusion of T cells. Nature. (2018) 554:544–8. doi: 10.1038/nature25501, PMID: 29443960 PMC6028240

[146] Vanpouille-Box C Diamond JM Pilones KA Zavadil J Babb JS Formenti SC. TGFβ Is a master regulator of radiation therapy-induced antitumor immunity. Cancer Res. (2015) 75:2232–42. doi: 10.1158/0008-5472.CAN-14-3511, PMID: 25858148 PMC4522159

[147] Bouquet F Pal A Pilones KA Demaria S Hann B Akhurst RJ. TGFβ1 inhibition increases the radiosensitivity of breast cancer cells *in vitro* and promotes tumor control by radiation *in vivo* . Clin Cancer Res. (2011) 17:6754–65. doi: 10.1158/1078-0432.CCR-11-0544, PMID: 22028490 PMC3724539

[148] Hardee ME Marciscano AE Medina-Ramirez CM Zagzag D Narayana A Lonning SM. Resistance of glioblastoma-initiating cells to radiation mediated by the tumor microenvironment can be abolished by inhibiting transforming growth factor-β. Cancer Res. (2012) 72:4119–29. doi: 10.1158/0008-5472.CAN-12-0546, PMID: 22693253 PMC3538149

[149] De Roock W Claes B Bernasconi D De Schutter J Biesmans B Fountzilas G. Effects of KRAS, BRAF, NRAS, and PIK3CA mutations on the efficacy of cetuximab plus chemotherapy in chemotherapy-refractory metastatic colorectal cancer: a retrospective consortium analysis. Lancet Oncol. (2010) 11:753–62. doi: 10.1016/S1470-2045(10)70130-3, PMID: 20619739

[150] Laurent-Puig P Cayre A Manceau G Buc E Bachet J-B Lecomte T. Analysis of PTEN, BRAF, and EGFR status in determining benefit from cetuximab therapy in wild-type KRAS metastatic colon cancer. J Clin Oncol Off J Am Soc Clin Oncol. (2009) 27:5924–30. doi: 10.1200/JCO.2008.21.6796, PMID: 19884556

[151] Jhawer M Goel S Wilson AJ Montagna C Ling Y-H Byun D-S. PIK3CA mutation/PTEN expression status predicts response of colon cancer cells to the epidermal growth factor receptor inhibitor cetuximab. Cancer Res. (2008) 68:1953–61. doi: 10.1158/0008-5472.CAN-07-5659, PMID: 18339877 PMC3972216

[152] Perrone F Lampis A Orsenigo M Di Bartolomeo M Gevorgyan A Losa M. PI3KCA/PTEN deregulation contributes to impaired responses to cetuximab in metastatic colorectal cancer patients. Ann Oncol Off J Eur Soc Med Oncol. (2009) 20:84–90. doi: 10.1093/annonc/mdn541, PMID: 18669866

[153] Sartore-Bianchi A Martini M Molinari F Veronese S Nichelatti M Artale S. PIK3CA mutations in colorectal cancer are associated with clinical resistance to EGFR-targeted monoclonal antibodies. Cancer Res. (2009) 69:1851–7. doi: 10.1158/0008-5472.CAN-08-2466, PMID: 19223544

[154] Peng W Chen JQ Liu C Malu S Creasy C Tetzlaff MT. Loss of PTEN promotes resistance to T cell-mediated immunotherapy. Cancer Discov. (2016) 6:202–16. doi: 10.1158/2159-8290.CD-15-0283, PMID: 26645196 PMC4744499

[155] Song M Chen D Lu B Wang C Zhang J Huang L. PTEN loss increases PD-L1 protein expression and affects the correlation between PD-L1 expression and clinical parameters in colorectal cancer. PloS One. (2013) 8:e65821. doi: 10.1371/journal.pone.0065821, PMID: 23785454 PMC3681867

[156] Chida K Kawazoe A Kawazu M Suzuki T Nakamura Y Nakatsura T. A low tumor mutational burden and PTEN mutations are predictors of a negative response to PD-1 blockade in MSI-H/dMMR gastrointestinal tumors. Clin Cancer Res Off J Am Assoc Cancer Res. (2021) 27:3714–24. doi: 10.1158/1078-0432.CCR-21-0401, PMID: 33926917

[157] Wang Q Shi Yl Zhou K Wang L Yan Z Liu Y. PIK3CA mutations confer resistance to first-line chemotherapy in colorectal cancer. Cell Death Dis. (2018) 9:739. doi: 10.1038/s41419-018-0776-6, PMID: 29970892 PMC6030128

[158] Hu F Song D Yan Y Huang C Shen C Lan J. IL-6 regulates autophagy and chemotherapy resistance by promoting BECN1 phosphorylation. Nat Commun. (2021) 12:3651. doi: 10.1038/s41467-021-23923-1, PMID: 34131122 PMC8206314

[159] Liu B Liu Y Zhao L Pan Y Shan Y Li Y. Upregulation of microRNA-135b and microRNA-182 promotes chemoresistance of colorectal cancer by targeting ST6GALNAC2 via PI3K/AKT pathway. Mol Carcinog. (2017) 56:2669–80. doi: 10.1002/mc.22710, PMID: 28767179

[160] Skvortsova I Skvortsov S Stasyk T Raju U Popper B-A Schiestl B. Intracellular signaling pathways regulating radioresistance of human prostate carcinoma cells. Proteomics. (2008) 8:4521–33. doi: 10.1002/pmic.200800113, PMID: 18821526

[161] Zhu W Fu W Hu L. NVP-BEZ235, dual phosphatidylinositol 3-kinase/mammalian target of rapamycin inhibitor, prominently enhances radiosensitivity of prostate cancer cell line PC-3. Cancer Biother Radiopharm. (2013) 28:665–73. doi: 10.1089/cbr.2012.1443, PMID: 23768063

[162] Datta K Suman S Fornace AJ. Radiation persistently promoted oxidative stress, activated mTOR via PI3K/Akt, and downregulated autophagy pathway in mouse intestine. Int J Biochem Cell Biol. (2014) 57:167–76. doi: 10.1016/j.biocel.2014.10.022, PMID: 25449263 PMC4363107

[163] Codony-Servat J Cuatrecasas M Asensio E Montironi C Martínez-Cardús A Marín-Aguilera M. Nuclear IGF-1R predicts chemotherapy and targeted therapy resistance in metastatic colorectal cancer. Br J Cancer. (2017) 117:1777–86. doi: 10.1038/bjc.2017.279, PMID: 29123263 PMC5729466

[164] Yang C Song J Hwang S Choi J Song G Lim W. Apigenin enhances apoptosis induction by 5-fluorouracil through regulation of thymidylate synthase in colorectal cancer cells. Redox Biol. (2021) 47:102144. doi: 10.1016/j.redox.2021.102144, PMID: 34562873 PMC8476449

[165] Itatani Y Yamamoto T Zhong C Molinolo AA Ruppel J Hegde P. Suppressing neutrophil-dependent angiogenesis abrogates resistance to anti-VEGF antibody in a genetic model of colorectal cancer. Proc Natl Acad Sci U S A. (2020) 117:21598–608. doi: 10.1073/pnas.2008112117, PMID: 32817421 PMC7474657

[166] Jiang SS Xie YL Xiao XY Kang Z-R Lin X-L Zhang L. *Fusobacterium nucleatum*-derived succinic acid induces tumor resistance to immunotherapy in colorectal cancer. Cell Host Microbe. (2023) 31:781–797.e9. doi: 10.1016/j.chom.2023.04.010, PMID: 37130518

[167] Krug J Rodrian G Petter K Yang H Khoziainova S Guo W. *N*-glycosylation regulates intrinsic IFN-γ Resistance in colorectal cancer: implications for immunotherapy. Gastroenterology. (2023) 164:392–406.e5. doi: 10.1053/j.gastro.2022.11.018, PMID: 36402190 PMC10009756

[168] Bao Y Zhai J Chen H Wong CC Liang C Ding Y. Targeting m6A reader YTHDF1 augments antitumour immunity and boosts anti-PD-1 efficacy in colorectal cancer. Gut. (2023) 72:1497–509. doi: 10.1136/gutjnl-2022-328845, PMID: 36717220 PMC10359538

[169] Chenthamara D Subramaniam S Ramakrishnan SG Krishnaswamy S Essa MM Lin F-H. Therapeutic efficacy of nanoparticles and routes of administration. Biomater Res. (2019) 23:20. doi: 10.1186/s40824-019-0166-x, PMID: 31832232 PMC6869321

[170] Huang CZ Zhou Y Tong QS Duan Q-J Zhang Q Du J-Z. Precision medicine-guided co-delivery of ASPN siRNA and oxaliplatin by nanoparticles to overcome chemoresistance of colorectal cancer. Biomaterials. (2022) 290:121827. doi: 10.1016/j.biomaterials.2022.121827, PMID: 36228517

[171] Russo A Maiolino S Pagliara V Ungaro F Tatangelo F Leone A. Enhancement of 5-FU sensitivity by the proapoptotic rpL3 gene in p53 null colon cancer cells through combined polymer nanoparticles. Oncotarget. (2016) 7:79670–87. doi: 10.18632/oncotarget.13216, PMID: 27835895 PMC5346744

[172] He S Wang Y Qi J Chen H Zhou W. Co-delivery liposomes of irinotecan hydrochloride and curcumin in the synergistic treatment of colorectal cancer. J Drug Delivery Sci Technol. (2024) 98:105848. doi: 10.1016/j.jddst.2024.105848

[173] Chen Q Feng L Liu J Zhu W Dong Z Wu Y. Intelligent albumin–mnO2 nanoparticles as pH-/H2O2-responsive dissociable nanocarriers to modulate tumor hypoxia for effective combination therapy. Adv Mater. (2016) 28:7129–36. doi: 10.1002/adma.201601902, PMID: 27283434

[174] El Hallal R Lyu N Wang Y. Effect of cetuximab-conjugated gold nanoparticles on the cytotoxicity and phenotypic evolution of colorectal cancer cells. Molecules. (2021) 26:567. doi: 10.3390/molecules26030567, PMID: 33499047 PMC7865832

[175] Ni Q Zhang F Liu Y Wang Z Yu G Liang B. A bi-adjuvant nanovaccine that potentiates immunogenicity of neoantigen for combination immunotherapy of colorectal cancer. Sci Adv. (2020) 6:eaaw6071. doi: 10.1126/sciadv.aaw6071, PMID: 32206706 PMC7080439

[176] Donthireddy L Vonteddu P Murthy T Kwak T Eraslan R-N Podojil JR. ONP-302 nanoparticles inhibit tumor growth by altering tumor-associated macrophages and cancer-associated fibroblasts. J Cancer. (2022) 13:1933–44. doi: 10.7150/jca.69338, PMID: 35399717 PMC8990435

[177] Neetika Sharma M Thakur P Gaur P Rani GM Rustagi S. Cancer treatment and toxicity outlook of nanoparticles. Environ Res. (2023) 237:116870. doi: 10.1016/j.envres.2023.116870, PMID: 37567383

[178] Huang Q Ge Y He Y Wu J Tong Y Shang H. The application of nanoparticles targeting cancer-associated fibroblasts. Int J Nanomed. (2024) 19:3333–65. doi: 10.2147/IJN.S447350, PMID: 38617796 PMC11012801

[179] Ding S Hsu C Wang Z Natesh NR Millen R Negrete M. Patient-derived micro-organospheres (MOS) enable clinical precision oncology. Cell Stem Cell. (2022) 29:905–917.e6. doi: 10.1016/j.stem.2022.04.006, PMID: 35508177 PMC9177814

[180] Wang H Zhang H Wang Y Brown ZJ Xia Y Huang Z. Regulatory T-cell and neutrophil extracellular trap interaction contributes to carcinogenesis in non-alcoholic steatohepatitis. J Hepatol. (2021) 75:1271–83. doi: 10.1016/j.jhep.2021.07.032, PMID: 34363921 PMC12888775

[181] Zhang H Wang Y Onuma A He J Wang H Xia Y. Neutrophils extracellular traps inhibition improves PD-1 blockade immunotherapy in colorectal cancer. Cancers. (2021) 13:5333. doi: 10.3390/cancers13215333, PMID: 34771497 PMC8582562

[182] Xia Y He J Zhang H Wang H Tetz G Maguire CA. AAV-mediated gene transfer of DNase I in the liver of mice with colorectal cancer reduces liver metastasis and restores local innate and adaptive immune response. Mol Oncol. (2020) 14:2920–35. doi: 10.1002/1878-0261.12787, PMID: 32813937 PMC7607180

[183] Ren J He J Zhang H Xia Y Hu Z Loughran P. Platelet TLR4-ERK5 axis facilitates NET-mediated capturing of circulating tumor cells and distant metastasis after surgical stress. Cancer Res. (2021) 81:2373–85. doi: 10.1158/0008-5472.CAN-20-3222, PMID: 33687949 PMC8137664

